# A scoping review of resilience among transition-age youth with serious mental illness: tensions, knowledge gaps, and future directions

**DOI:** 10.1186/s12888-023-05158-0

**Published:** 2023-09-07

**Authors:** Amy E. Nesbitt, Catherine M. Sabiston, Melissa L. deJonge, Skye P. Barbic, Nicole Kozloff, Emily J. Nalder

**Affiliations:** 1 Rehabilitation Sciences Institute, University of Toronto, 500 University Avenue, Toronto, ON M5G 1V7 Canada; 2 Faculty of Kinesiology and Physical Education, University of Toronto, Toronto, ON Canada; 3 Department of Occupational Science and Occupational Therapy, University of British Columbia, Vancouver, BC Canada; 4 Foundry, Vancouver, BC Canada; 5 Providence Research, Vancouver, BC Canada; 6 Child, Youth and Emerging Adult Program, Centre for Addiction and Mental Health, Toronto, ON Canada; 7 Department of Psychiatry, University of Toronto, Toronto, ON Canada; 8 Department of Occupational Science and Occupational Therapy, University of Toronto, Toronto, ON Canada

**Keywords:** Adolescent, Young adult, Resilience, Mental health, Review, Advisory group

## Abstract

**Introduction:**

The study of resilience among transition-age youth (aged 16–29 years) living with serious mental illness (SMI) has provided a promising new direction for research with the capacity to explore individuals’ strengths and resources. However, variability in how resilience is defined and measured has led to a lack of conceptual clarity. A comprehensive synthesis is needed to understand current trends and gaps in resilience research among this population. The purpose of the current study was to map how resilience has been conceptualized and operationalized among transition-age youth with SMI, explore resilience factors and outcomes that have been studied, and recommend areas for future research.

**Methods:**

A six-stage scoping review methodology was used to systematically identify relevant empirical literature across multiple databases (MEDLINE, EMBASE, PsycINFO, AMED, CINAHL, Scopus), addressing transition-age youth diagnosed with SMI and resilience. Topic consultation and reaction meetings were conducted to gather feedback from transition-age youth with SMI, researchers, and clinicians during the review process to enhance the applicability of the review findings. A meta-narrative approach was used to organize included studies into research traditions (i.e., paradigms of inquiry with similar storylines, theoretical and methodological orientations). Resilience factors and outcomes, and the consultative meetings, were analyzed using content analysis.

**Results:**

Twenty-four studies met inclusion criteria (14 quantitative, 9 qualitative, 1 mixed-method). Four research traditions were identified, each contributing a unique storyline which conceptualized and operationalized resilience in slightly different ways: Stress Adaptation, Person-Environment Interactions, Recovery-Focused, and Critical and Cultural Perspectives. Resilience factors and outcomes were most commonly evaluated at the individual-level or within the immediate environment (e.g., personal characteristics, social support networks). Limited research has explored the influence of macro-level systems and health inequalities on resilience processes. Results from the consultative meetings further demonstrated the importance of health services and sociocultural factors in shaping processes of resilience among youth.

**Conclusion:**

The present results may be used to inform future work, as well as the development of age-appropriate, strengths-based, and resilience-oriented approaches to service delivery. Interdisciplinary and intersectional research that prioritizes community and youth engagement is needed to advance current understandings of resilience among transition-age youth with SMI.

**Supplementary Information:**

The online version contains supplementary material available at 10.1186/s12888-023-05158-0.

## Background

Over 20% of transition-age youth (age 16–29 years old) are living with serious mental illness (SMI), defined as mental health conditions that cause substantial disruptions to everyday functioning (e.g., depression, bipolar disorder, schizophrenia spectrum disorders) [1, 2]. The onset of SMI for transition-age youth occurs during a critical period of development, characterized by increased independence and responsibility in social and occupational roles, identity formation, and many complex life transitions [3]. Consequently, the experience of SMI can have a pronounced impact on young people’s developmental trajectory, quality of life, physical health, and community engagement [4–6]. Aligning with a shift towards strengths-based, recovery-oriented, and early intervention service approaches over the past two decades, there has been a surge of research focused on the concept of *resilience* among transition-age youth [7, 8]. Broadly, resilience refers to positive adaptation in the face of significant adversity and is considered an important component of one’s personal recovery process [8, 9]. Finding new pathways and services that foster young people’s recovery and resilience is a crucial priority in recent national and global mental health strategies [10–12].

### Resilience among transition-age youth

The study of resilience provides a unique framework for understanding the complex personal factors and systems that contribute to youth mental health. Particularly, adopting a resilience perspective may re-frame and de-pathologize conceptions of youth SMI, turning our attention towards one’s strengths, values, and resources, in contrast to a sole focus on individual risks or impairment [13, 14]. Additionally, a focus on building transition-age youth resilience may inform transdiagnostic models of care by identifying protective factors and mechanisms that foster positive indices of development and well-being among young people with diverse experiences and diagnoses [15–18].

Importantly, the concept of resilience has evolved over time, from early views of resilience as an exceptional or fixed trait within an individual [19], to more recent research applying a process-oriented perspective [20, 21]. From this point of view, resilience is considered a complex, fluid, and malleable process that unfolds over time, encompassing both aspects unique to the individual (e.g., personal qualities) and wider social-ecological features of their environment (e.g., one’s social support network and cultural context) [18, 22, 23]. Recent work adopting this process-oriented perspective has begun to address critiques to the study of resilience as potentially placing increased pressure or responsibility on young people to simply “be resilient”, without enough emphasis on the sociocultural and systemic conditions that contribute to resilience via person-environment interactions [24, 25]. This has also sparked new research among transition-age youth with SMI exploring a wide range of biological and psychosocial protective factors in connection to clinical outcomes, the effectiveness of resiliency-informed interventions, and environmental circumstances that facilitate resilience processes during this transitional stage of life [26–28].

While a resilience approach clearly shows value and promise to understanding transition-age youth’s experience of SMI, there remains a lack of clarity on the meanings, processes, and outcomes of resilience among this population. This is, in part, due to the large variety of ways in which resilience has been conceptualized within the youth mental health literature, as this directly impacts the research questions that are addressed, and how the concept is understood, operationalized, and applied [29]. Additionally, there is no single resilience theory or model specifically tailored to the unique experiences of transition-age youth living with SMI to guide further research and practice [7]. Prior reviews have synthesized the evolution of resilience theory, measures, and outcomes, as well as the wide range of biological, psychosocial, environmental and cultural factors that are theorized to influence resilience among youth and adults [18, 30–34]. None of these focused specifically on transition-age youth with SMI. As such, current trends and gaps within resilience research among this population remain unclear. Additionally, researchers have argued that engaging community members through consultations and/or partnerships is an imperative step in resilience research to improve current practices and avoid “de-contextualizing” youth’s experiences [35]. This is an important limitation to recent reviews of resilience literature undertaken among broader youth and adult populations [30–34]. Integrating the perspectives of youth, clinicians, and researchers can provide valuable insights to the study of resilience and improve the applicability and uptake of research findings. Taken together, a comprehensive synthesis of existing research is needed to explore how the concept of resilience has been studied among transition-age youth with SMI.

### Present investigation

A scoping review of published research from the last 22 years was conducted to enhance conceptual clarity in this area, identify factors and outcomes that are relevant to transition-age youth’s resilience, and recommend areas for future research. The review was informed by community advisory group consultations, a meta-narrative approach, and current process-oriented models of resilience within rehabilitation sciences.

A meta-narrative review approach [36] was used to map how conceptualizations of resilience have evolved over time and across different research traditions. According to Greenhalgh et al. (2005), research traditions are considered paradigms of inquiry that share similar theoretical orientations, methodological approaches, conceptual papers, and perspectives, which are portrayed through an overarching storyline or lens. Meta-narrative review is recommended for synthesizing complex, heterogeneous bodies of literature where a key concept has evolved over time and conceptual clarity is needed. This approach is particularly useful for exploring potential tensions and knowledge gaps that exist across research traditions [36].

McLarnon and Rothstein’s (2013) conceptual model of resilience [20] and Nalder et al.’s (2019) traumatic brain injury resiliency model [21] also guided the focus and scope of the present investigation. These models provided a framework for exploring the transactional nature of multi-modal resilience factors and processes believed to contribute to resilience over time. Resilience processes are depicted as the subjective experience of how individuals negotiate and “bounce back” from adversities within their specific context. Based on these process-oriented models [20, 21], the following core elements of resilience make up the focus of the current review: (1) adversity, (2) personal characteristics, (3) environmental resources, (4) self-regulatory strategies, and (5) resilience-related outcomes (see Table 1 for detailed descriptions).

**Table 1:** PCC criteria defining the scope of the current review

PCC Element	Definition
**Population** Age SMI	Transition-age youth who have experienced SMI Middle adolescence (age 15) to the “upper limit” of young adulthood (age 36) [3, 37, 38] Mental illnesses that cause substantial functional impairment (e.g., major depressive disorders, bipolar disorders, personality disorders, anxiety disorders, eating disorders and schizophrenia spectrum disorders) [39, 40]
**Concept** Resilience Core elements	Resilience, including five core elements [20, 21] “A dynamic process that unfolds over time, involving multiple resilience factors that interact to enable individuals to negotiate or recover from stressful life events / adversity” [41] (1) Adversity: subjective experiences of stress, hardships, trauma, challenges, or other adverse circumstances. (2) Personal characteristics: internal protective / risk factors reflected as individual traits or qualities. (3) Environmental resources: external protective / risk factors reflected as social supports, services, resources, or social determinants of health. (4) Self-regulatory strategies / processes: the strategies and mechanisms through which young people self-manage their mood, emotions, thoughts, and/or behaviors. (5) Resilience-related outcomes: indices of positive development, adaptation, health, well-being etc
**Context**	Research conducted in any individual, community, or health-oriented setting that may reflect the context of transition-age youth’s personal mental health recovery

## Methods

The scoping review process followed an established six-stage method [42, 43]. Guidelines and criteria for conducting and reporting scoping reviews were applied [44] (see online supplementary file 1 for the PRISMA-ScR checklist), as well as recent recommendations for meta-narrative review [45] and engaging community advisory groups [46]. An iterative and team-based approach was used with frequent meetings among the multidisciplinary review team, incorporating multiple perspectives while refining the research design and results. A protocol was developed and registered in advance [41].

### Stage 1: identifying the research question

The scoping review was guided by two research questions: (1) How has resilience been conceptualized and operationalized (i.e., defined and measured) in the transition-age youth mental health literature? (2) What factors influence resilience among transition-age youth with SMI, and what outcomes have been studied within the context of transition-age youth’s mental health recovery? Questions were specifically developed to address the population, concept, and context of interest (PCC mnemonic) [47]. Each component was defined a priori in the protocol [41] and are shared in Table 1 to clearly define the scope of the review.

### Stage 2: identifying relevant literature

A multi-database search strategy was developed in consultation with a health sciences librarian at the University of Toronto (see online supplementary file 2). Six electronic databases were searched to systematically identify relevant empirical studies: MEDLINE, EMBASE, PsycINFO, AMED, CINAHL, and Scopus. Specified search terms were explored using keywords and controlled vocabulary (subject / MeSH headings) and combined with appropriate Boolean logic. The search strategy was peer reviewed by two experienced mental health researchers external to the review team using the CADTH Peer Review Checklist for Search Strategies [48] before being conducted on December 6, 2021. Additional sources were identified by manually searching the reference lists of relevant reviews and the included articles.

### Stage 3: study selection

Search results were exported from each database to Endnote to remove duplicate files. All search results were then transferred to an online systematic review software (Covidence) for data management and screening. Articles were screened in duplicate (AEN, MLdJ) using predetermined eligibility criteria defined for two stages of screening: i) title / abstract, and ii) full-text review. Specific inclusion and exclusion criteria were piloted at each screening stage using a subset of 10 randomly selected articles until 80% agreement was met. Disagreements were resolved by discussion or the decision of a third reviewer. Challenges and uncertainties were also brought to the attention of the four content experts (CMS, SPB, NK, EJN). For inclusion in this scoping review, articles were required to meet the following criteria [41]: a) Population: Referred to transition-age youth diagnosed or living with SMI. b) Concept: Clearly defined or operationalized the concept of resilience from a process-oriented perspective. c) Type of source: Contained peer reviewed original research (quantitative, qualitative, mixed-method). d) Publication language / date: Written in English and published between 2000 and 2022. The publication date of included articles was limited to the year 2000 onwards (~ 22-year period) given two trends that emerged during this time: greater adoption of process-oriented perspectives of resilience within mental health research [7, 8], and increased focus on the developmental period of transition-age youth and the evaluation of mental health services for this population (e.g., early intervention programs) [1, 38].

### Stage 4: data extraction

A standardized charting form was used to extract, organize, and interpret data from the relevant articles. (1) General document details and study characteristics included the APA citation, country, study context, and academic discipline. (2) Participant characteristics included age, SMI diagnosis, age at onset of SMI, stage of illness, demographic information, and sample size. (3) Informed by meta-narrative review [36], the following details were extracted to capture the interrelated dimensions that are shared within a research tradition (conceptual, theoretical, methodological, instrumental): (a) study purpose and research questions / objectives, (b) theoretical frameworks / models applied, (c) conceptualizations and definitions of resilience, (d) study design and methods (e.g., main methods used, intervention characteristics, intersectional approaches, type of youth engagement) [49], (e) resilience measures. (4) Informed by process-oriented models of resilience [20, 21], explanatory variables (e.g., predictors, mediators, moderators) and key constructs (e.g., qualitative themes) that were emphasized and directly linked to resilience-related outcomes were extracted and sorted in accordance with the five core elements of resilience: (a) adversity, (b) personal characteristics, (c) environmental resources, (d) self-regulatory strategies, and (e) resilience-related outcomes. (5) Key messages and important results were also extracted to supplement the data above and support our interpretation of the relevance of each paper for this review [36].

One reviewer (AEN) completed extraction for all studies. A second reviewer (MLdJ), who assisted in the initial development of the charting form, verified a subset of articles (25%) to ensure consistency in data extraction. Challenges and uncertainties throughout this stage of the review were discussed with the rest of the review team (CMS, SPB, NK, EJN), who have research and clinical expertise in young adult mental health and resiliency.

### Stage 5: collating, summarizing, and reporting the results

To address the first research question, a meta-narrative approach was used to synthesize how resilience has been conceptualized and operationalized within the transition-age youth mental health literature [36]. Findings were described by mapping conceptualizations of resilience over time and across different research traditions (or “paradigms”). The identification of research traditions involved grouping articles that reflected a similar conceptual focus (e.g., purpose, key variables), theoretical orientation (e.g., resilience frameworks, theorists, definitions), and methodological / instrumental approach (e.g., study design, measures), and by considering how resilience was portrayed as an overarching storyline or ‘lens’ [36]. This information was coded using an inductive and iterative process until preliminary research traditions could be generated, each demonstrating a unique narrative. Research traditions were then further refined, with increased focus on analyzing temporal trends, re-visiting and cross-referencing information in the original articles, and continuously comparing each study and tradition [36, 45].

To address the second research question, qualitative content analysis [50, 51] was used to identify types of resilience factors and outcomes that have been studied among transition-age youth diagnosed with SMI. Descriptions of the explanatory / outcome variables and qualitative themes extracted in the charting form were inductively analyzed through a process of open-coding and then grouping variables into subcategories. The coding and abstraction process was guided by two process-oriented models [20, 21], allowing for further grouping of subcategories that aligned with the five core elements of resilience (adversity, personal characteristics, environmental resources, self-regulatory strategies, resilience-related outcomes). This was an ongoing, interpretive process, whereby some of the variables extracted were re-categorized based on new interpretations, emerging patterns, and the creation of higher order headings. Bronfenbrenner's (1979) ecological systems theory informed the analysis and interpretation of internal and external protective factors identified [52]. Lastly, frequencies (%) and counts (n) were used to synthesize key study characteristics, and to supplement the narrative descriptions throughout. Meta-narrative and content analyses were conducted by one reviewer (AEN). Preliminary analyses to identify research traditions and categorize resilience factors and outcomes were reviewed by a second reviewer (MLdJ) who acted as a critical friend by discussing, verifying and challenging interpretations from a critical perspective [53]. All members of the review team then refined the analyses and results through multiple discussions.

### Stage 6: community advisory group consultation

Guided by recent recommendations [46], this scoping review engaged community advisory groups throughout the review process to enhance the relevance and applicability of the review findings. Following approval by the University of Toronto Health Sciences Research Ethics Board (REB #: 42495), transition-age youth with lived experience of SMI, clinicians, and mental health / resilience researchers were invited to participate in consultative meetings at two time points: topic consultation and input meetings (before completing study selection), and reaction meetings (after synthesizing included studies). Participants provided informed consent before completing a brief demographic questionnaire and participating in a focus group.

Topic consultation and input meetings focused on discussing the scoping review protocol and participants were asked to share their perspectives of youth resilience, what they would most like to learn from the review, and feedback on the review objectives and methods. During the reaction meetings, the results of the review were shared, and participants discussed their overall impression of the findings, recommendations for future research, and how this knowledge may be used or applied. Preferences for knowledge dissemination were collected at both time points. One focus group was conducted with transition-age youth at each time point (input meeting *n* = 6; reaction meeting *n* = 5), and one focus group was conducted with researchers and clinicians at each time point (input meeting *n* = 4; reaction meeting *n* = 7), resulting in a total of four consultative meetings (*N* = 20; one researcher and one transition-age youth participant attended both input and reaction meetings). Focus group discussions were 75–90 min in duration.

Two members of the review team (AEN, MLdJ) co-facilitated the focus groups virtually using a videoconferencing platform (Zoom) and semi-structured interview guides. All focus groups were audio recorded, transcribed verbatim, and analyzed inductively using qualitative content analysis [50, 51]. Analyses began with multiple readings of the transcripts to gain a holistic sense of the data and reviewing the field notes from each focus group discussion. Analyses were conducted using Nvivo software and followed a process of open-coding, the creation of initial categories and higher order headings, and abstraction. Findings were reported to capture advisors’ perspectives and feedback with exemplary quotes. One reviewer (AEN) led the analyses, developed the preliminary findings, and then sought feedback from a second reviewer, who reviewed and critiqued initial interpretations in the role of a critical friend (MLdJ) [53]. Findings were then discussed in depth among the entire review team.

### Transparency and rigor

Specific methods were employed to enhance the trustworthiness and rigor of the review process. Results were synthesized and reported in line with the PRISMA-ScR Checklist [44]. A detailed audit trail was used to track important decisions among the review team [54]. Ongoing reflexive practice was also used to acknowledge how our unique positions, backgrounds, and experiences may contribute to pre-existing assumptions about youth mental health and resilience, and thus impact methodological choices, analyses, and interpretations. Reflexivity encouraged the review team to consider and confront potential biases and power differentials during the research activities [45, 53].

## Results

**Fig. 1:**
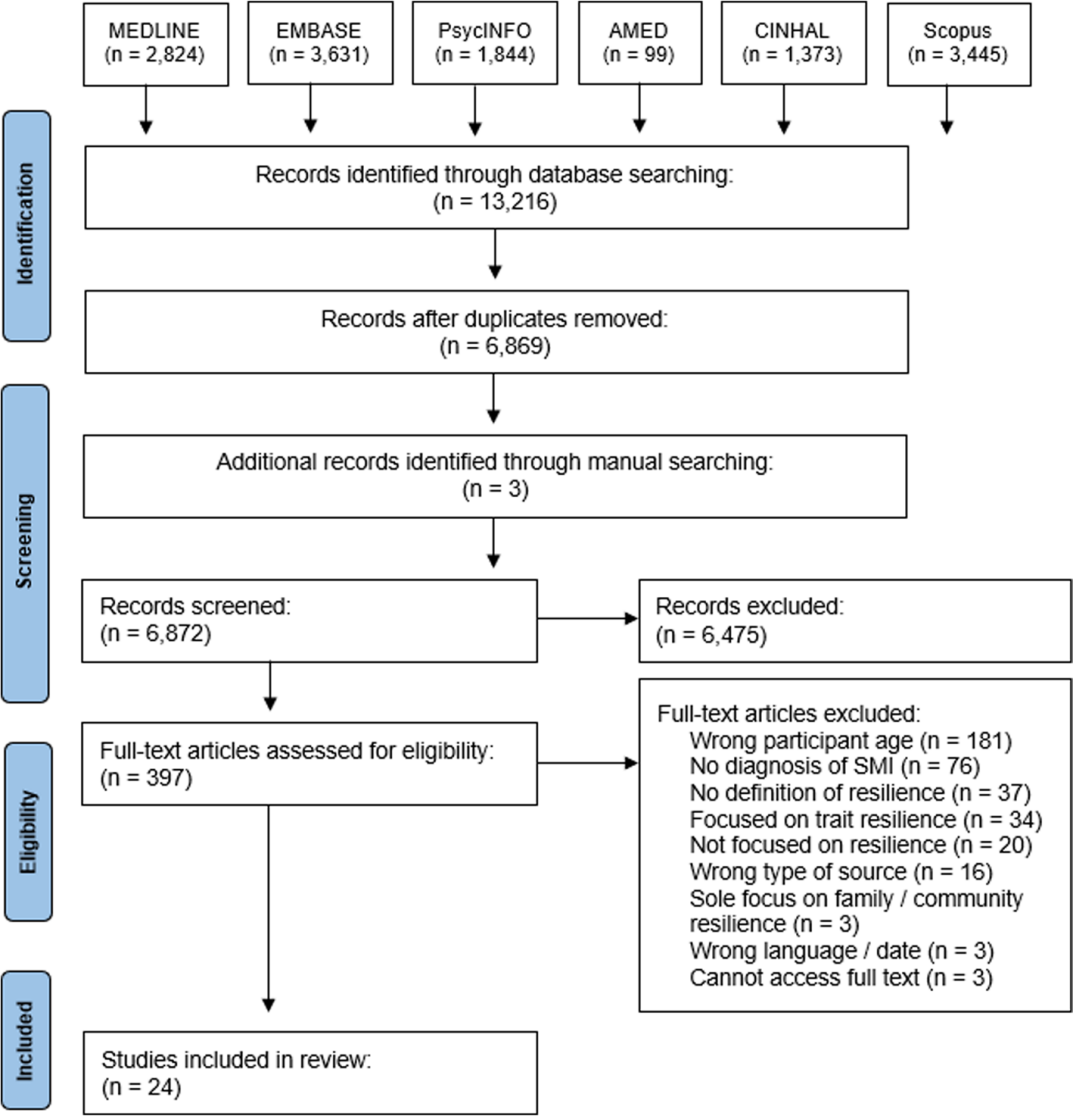
PRISMA flow diagram for scoping reviews

The initial search identified 6,872 unique articles following the removal of duplicates. Following the 2-stage screening procedures, 397 full-texts were reviewed for eligibility. A total of 24 published articles met all inclusion criteria and were included in this review (see Fig. 1 for the PRISMA flow diagram).

### Study characteristics

The 24 included studies were published between 2003 and 2021, and were from diverse geographic locations, including the U.S.A. (*n* = 7), Australia (*n* = 3), Brazil (*n* = 3), Canada (*n* = 2), Korea (*n* = 2), Italy (*n* = 1), The Netherlands (*n* = 1), New Zealand (*n* = 1), Norway (*n* = 1), South Africa (*n* = 1), Spain (*n* = 1), and Switzerland (*n* = 1). Based on the author affiliations and publication journal, the academic disciplines of the included articles were: psychiatry (*n* = 12), psychology (*n* = 7), nursing (*n* = 2), medicine (*n* = 2), and interdisciplinary (*n* = 1). Study and participant characteristics are summarized in Table 2.

**Table 2:** Details of included studies (*N* = 24) exploring resilience among transition-age youth with SMI

Author	Country	Participant Characteristics: Diagnosis (Dx), Onset of SMI, Age (Range, Mean ± SD), Sex/Gender (%), Race/Ethnicity (%)	Sample Size (N)	Academic Discipline
Seok et al. (2012) [55]	Korea	**Dx:** Major Depressive Disorder (MDD). **Onset:** n/r **Age:** M = 31.9 ± 1.8 years. **Sex/Gender:** 73.1% Female. **Race/Ethnicity:** n/r	***N* = **26	Psychiatry
Fischer et al. (2018) [56]	U.S.A	**Dx:** Major Depressive Disorder (MDD). **Onset:** n/r **Age:** M = 18.9 ± 2.5 years. **Sex/Gender:** 100% Female. **Race/Ethnicity:** 65% White	***N* = **20	Psychiatry
Konradt et al. (2018) [57]	Brazil	**Dx:** Major Depressive Disorder (MDD). **Onset:** n/r **Age:** 18–29 years (M = 23.98 ± 3.38). **Sex/Gender:** 75.8% Women. **Race/Ethnicity:** n/r	***N* = **61	Psychiatry
De Berardis et al. (2020) [58]	Italy	**Dx:** Major Depression. **Onset:** Young adulthood **Age:** 18–37 years (M = 25.2 ± 3.8). **Sex/Gender:** n/r. **Race/Ethnicity:** n/r	***N* = **103	Psychiatry
Vieira et al. (2020) [59]	Brazil	**Dx:** Major Depressive Disorder (MDD), Bipolar Disorder (BD). **Onset:** n/r **Age:** MDD: M = 26.02 ± 2.13 years; BD: M = 25.78 ± 2.11 years **Sex/Gender:** 75.2% Female. **Race/Ethnicity:** 64.1% White	***N* = **407	Psychiatry
Peters et al. (2021) [60]	Brazil	**Dx:** Major Depressive Disorder (MDD). **Onset:** n/r **Age:** 18–29 years. **Sex/Gender:** 78.3% female. **Race/Ethnicity:** n/r	***N* = **106	Psychiatry
Fergusson et al. (2003) [61]	New Zealand	**Dx:** Major Depression. **Onset:** Young adulthood **Age:** 14–21 years. **Sex/Gender:** 49.8% Female. **Race/Ethnicity:** n/r	***N* = **403	Psychology
Gralinski-Bakker et al. (2004) [26]	U.S.A	**Dx:** Any clinically diagnosed SMI, defined as serious psychiatric disorder requiring inpatient hospitalization for 2–12 months. **Onset:** Adolescence (M = 14.4 years) **Age:** T1: M = 25.8 years; T2: 26–35 years (30.35 ± 2.26) **Sex/Gender:** 53% Women. **Race/Ethnicity:** n/r	***N* = **49	Psychology
Hauser et al. (2007) [62]	U.S.A	**Dx:** Any clinically diagnosed SMI, defined as serious psychiatric disorder requiring inpatient hospitalization for 2–12 months. **Onset:** Adolescence (13–16 years old) **Age:** T1: 14–17 years; T2: young adulthood (18 +).^a^ **Sex/Gender:** 44% Female. **Race/Ethnicity:** Predominantly Caucasian	***N* = **67	Psychology
Tan et al. (2015) [63]	Australia	**Dx:** Any axis 1 mental health disorder. Mixed mental disorders and clinically relevant decline in functioning. **Onset:** Adolescence **Age:** 13–18 years (15.40 ± 1.55). **Sex/Gender:** 75% Female. **Race/Ethnicity:** n/r	***N* = **80	Psychiatry
Marvin et al. (2017) [64]	U.S.A	**Dx:** Any clinically diagnosed SMI requiring residential treatment. 31% also had a learning disability. **Onset:** n/r **Age:** M = 14.85 ± 1.78 years. **Sex/Gender:** 100% Girls. **Race/Ethnicity:** 58% Caucasian	***N* = **36	Psychology
Hauber et al. (2019) [65]	The Netherlands	**Dx:** Personality Disorders. > 50% with co-occurring axis 1 disorder(s). **Onset:** n/r **Age:** 16–23 years (M = 18.9 ± 1.7). **Sex/Gender:** 88.6% Female. **Race/Ethnicity:** n/r	***N* = **70	Psychiatry
Hadebe et al. (2020) [66]	South Africa	**Dx:** Any SMI (schizophrenia, depression, bipolar disorder, anxiety).^b^**Onset:** n/r **Age:** 19–34 years. **Sex/Gender:** 30% Female. **Race/Ethnicity:** n/r Young adults living in a low-resource area	***N* = **10	Nursing
Gårdvik et al. (2021) [67]	Norway	**Dx:** Primary diagnosis of mood disorder or anxiety disorder. Former outpatients with high degree of comorbidity and complex symptom patterns. **Onset:** n/r **Age:** T1: 13–18 years (M = 15.7 ± 1.7); T2: 16–21 years (M = 18.5 ± 1.6) **Sex/Gender:** 56.8% Girls. **Race/Ethnicity:** n/r	***N* = **254	Medicine
Zimmermann et al. (2021) [68]	Switzerland	**Dx:** Borderline Personality Disorder (BPD). **Onset:** n/r **Age:** M = 16.6 ± 1.5 years. **Sex/Gender:** 100% Female. **Race/Ethnicity:** n/r	***N* = **15	Psychology
Henderson et al. (2015) [69]	Australia	**Dx:** First Episode of Psychosis (FEP). **Onset:** Within the past 36 months **Age:** 19–28 years. **Sex/Gender:** 70% Male. **Race/Ethnicity:** n/r	***N* = **10	Psychiatry
Las Hayas et al. (2016) [70]	Spain	**Dx:** Eating Disorder (e.g., anorexia nervosa (AN), bulimia nervosa (BN), both AN & BN, eating disorders not otherwise specified). **Onset:** Adolescence (M = 16.6 ± 3.7 years) **Age:** M = 35.6 ± 6.7 years.^a^**Sex/Gender:** 100% Women. **Race/Ethnicity:** n/r	***N* = **20	Psychology
Grob et al. (2020) [71]	U.S.A	**Dx:** Depression. 47% had co-occurring mental health conditions. **Onset:** Adolescence (< 15 years) to emerging adulthood (≥ 15 years) **Age:** 18–29 years. **Sex / Gender:** 50% Female. **Race / Ethnicity:** 63.9% White Participants recruited for maximum diversity (e.g., social identities, geographic locations)	***N* = **38	Medicine
Luther et al. (2020) [72]	U.S.A	**Dx:** Schizophrenia or Bipolar Disorder with Current Psychosis Early psychosis group: individuals < 36 years old. **Onset:** M = 19.90 ± 4.49 years **Age:** M = 25.47 ± 4.47 years. **Sex/Gender:** 63% Male. **Race/Ethnicity:** 53% African American	***N* = **30	Psychiatry
Delman et al. (2017) [73]	U.S.A	**Dx:** n/r. **Onset:** n/r. Young adults in recovery from “serious mental health conditions” **Age:** 21–26 years (M = 24). **Sex/Gender:** 57% Female. **Race/Ethnicity:** 100% White	***N* = **7	Psychiatry
Lal et al. (2017) [74]	Canada	**Dx:** Schizophrenia Spectrum and Affective Psychoses. **Onset:** Within the past 3 years **Age:** 18–24 years (M = 22) **Sex/Gender:** 71% Male. **Race/Ethnicity:** 41% First Nations, Asian, and Latin American Participants from diverse sociocultural and economic backgrounds	***N* = **17	Interdisciplinary^c^
Rayner et al. (2018) [75]	Australia	**Dx:** Any SMI, defined as a lifelong psychiatric condition that substantially disrupts daily functioning. All participants reported one or more co-occurring disorders (e.g., anxiety, depression, schizophrenia, bipolar, borderline personality disorder). **Onset:** n/r **Age:** 18–23 years (M = 20). **Sex/Gender:** 66.7% Female. **Race/Ethnicity:** n/r	***N* = **15	Psychology
Shalanski et al. (2019) [14]	Canada	**Dx:** Any SMI (e.g., PTSD, depression, and addiction). History of complex mental health problems and trauma. **Onset:** n/r **Age:** 15–16 years. **Sex/Gender:** 100% girls. **Race/Ethnicity:** n/r	***N* = **5	Nursing
Kim et al. (2020) [76]	Korea	**Dx:** Psychosis and Schizophrenia Spectrum Disorders. **Onset:** n/r **Age:** Acute stage: M = 28.7 ± 8.7 years; Stabilization phase: M = 26.6 ± 7.0 years.^b^ **Sex / Gender:** 45% Women. **Race / Ethnicity:** n/r	***N* = **340	Psychiatry

Studies are listed in accordance with the research traditions identified in Table 3 (rather than alphabetically / chronologically). n/r, not reported

^a^Broader age range considered acceptable for inclusion in this review based on retrospective study design [62, 70] and relevance to the study of transition-age youth and early intervention [76]

^b^Included one participant with primary diagnosis of epilepsy [66]

^c^Multiple academic disciplines identified (rehabilitation, social work, psychiatry, occupational therapy, education). T1 and T2: used to indicate measures at multiple time points (e.g., baseline and follow up)

Included studies often involved transition-age youth with mixed or any SMI (41.6%), however many studies focused on specific diagnoses, including major depressive disorder (29.2%), schizophrenia spectrum disorders / psychosis (16.7%), personality disorders (8.3%), and eating disorders (4.2%). Due to the inclusion of studies using retrospective methods, research participants ranged in age from 13–37 years old. Mean age ranged from 14.9 years (middle adolescence) to 35.6 years (young adulthood). Ten studies reported the age at onset of SMI as occurring during adolescence, young adulthood, or both (onset was not clearly reported in 14 articles). Six studies focused on experiences of youth navigating a first episode [58, 61, 69, 72, 74, 76], four studies focused on chronic / recurring SMI [26, 62, 71, 75], and three studies described participants as “recovered” or in recovery [56, 70, 73] (stage of illness was not clearly identified in 11 sources). Based on the 23 studies that reported sex and/or gender, nearly half involved participants identifying as predominantly female (29.2%), or girls / women (16.6%). Race and/or ethnicity was not reported in sixteen articles. Based on the available evidence, most samples were predominantly White/Caucasian; however, 20.8% of the studies involved more diverse samples with greater representation of youth from racialized or minority groups.

### RQ1: HOW has resilience been conceptualized and operationalized?

Meta-narrative review was used to map how the concept of resilience has been conceptualized and operationalized among transition-age youth with SMI. Four research traditions were identified, each contributing a unique storyline or ‘lens’ to the study of resilience: Stress Adaptation (*n* = 6), Person-Environment Interactions (*n* = 9), Recovery-Focused (*n* = 4), and Critical and Cultural Perspectives (*n* = 5). Key features of the articles included within each research tradition are presented in Table 3.

**Table 3:** Overview of each research tradition, detailing key shifts in the theoretical orientations, conceptual focus, and methodological approaches applied to the study of resilience among transition-age youth with SMI

**Research Traditions**		**Theoretical Orientation**	**Conceptual Focus**	**Methodological / Instrumental Approach**	
**Overview & Approach**	**Author**	**Definition**	**Purpose**	**Study Design**	**Operationalization & Measures**
**Stress Adaptation** **(*n* = 6)** Guiding Frameworks: Integrative frameworks of stress [77–80] Main resilience theorists: Rutter (interactive) [81], Masten (developmental) [82] Conceptualized resilience as: A process of positive adaptation determined through integrated biological, psychological, social, and environmental factors Focus: Emphasized personal protective factors that promote adaptation Approach: Variable-centred	Seok et al. (2012) [55]	The personal characteristics that enable one to adapt to environmental challenges and to overcome adversities or stressors	To investigate the relationships between early life stress and resilience factors with depressive symptom severity	Quantitative Cross-sectional study	Personal **resilience factors** CD-RISC [83] 5 Subscales: self-efficacy, self-confidence, optimism, self-control, spirituality / autonomy
	Fischer et al. (2018) [56]	The process of adapting well in the face of significant sources of stress and bouncing back from difficult life experiences	To investigate neural markers of resilience to depression, and the modulatory role of positive / negative life events	Quantitative Longitudinal study	Personal (neurobiological) **resilience factors**
	Konradt et al. (2018) [57]	The ability to adapt successfully in the face of stress and adversity, maintaining normal psychological and physical functioning	To assess the effects of resilience on severity of depressive and anxious symptoms after psychotherapy	Quantitative Clinical follow-up study nested in a randomized clinical trial	A **mechanism** and an **outcome** RS [84] Global Score
	De Berardis et al. (2020) [58]	The adaptive ability to cope with adversity or trauma	To examine the relationships between alexithymia, somatic sensations, resilience, and suicidal ideation	Quantitative Cross-sectional study	Personal **resilience factors** CD-RISC [83] Global Score
	Vieira et al. (2020) [59]	The ability to maintain relatively healthy and stable levels of physical and psychological functioning in the wake of traumatic experiences	To examine the mediating effect of resilience on the relationship between childhood trauma and mood disorder / depressive symptom severity	Quantitative Cross-sectional study	A **mechanism** RS [84] Global Score
	Peters et al. (2021) [60]	A person’s ability to adapt successfully to acute stress, trauma, or chronic forms of adversity	To explore clinical and biological correlates of resilience, and differences in therapeutic effects based on genetic markers	Quantitative Randomized clinical trial ^a^SGBA	Personal (genetic) **resilience factors**, and an **outcome** RS [84] Global Score
**Person-Environment Interactions (*n* = 9)** Guiding Frameworks: Psychosocial theories (narrative medicine [85], social-emotional learning framework [86], tripartite model of depression and anxiety [87], Yalom’s 12 therapeutic factors [88], neurodevelopmental model of resilience [89]) Main resilience theorists: Rutter (interactive) [81, 90, 91], Masten (developmental) [82, 92, 93], Luthar (multidimensional) [22] Conceptualized resilience as: A complex and transactional process dependent on both personal and environmental factors Focus: Increased emphasis on external protective factors and transactional processes between each person and their immediate environment Approach: Variable-centred Person-centred Life-course	Fergusson et al. (2003) [61]	A set of protective factors that may mitigate risk of developing suicidal behaviors	To explore factors that may contribute to vulnerability or resiliency to suicidal behaviors among young people	Quantitative Longitudinal cohort study ^a^SGBA	Personal / environmental (individual, familial, school, peer-related) **resilience factors**
	Gralinski-Bakker et al. (2004) [26]	Successful adaptation among individuals who faced challenging or threatening circumstances	To examine early adult indicators of psychosocial adjustment as predictors of adult markers of resilience (functioning and well-being) over time	Quantitative Longitudinal study ^a^SGBA	A **process** over time, and personal / environmental **resilience factors**
	Hauser et al. (2007) [62]	Unexpected adaptation in the face of serious adversity	To understand how resilient development unfolds among young people who have experienced SMI, and how protective processes change over the life-course	Qualitative Narrative follow-back study	A **process** over time, and “resilient functioning” as an **outcome**
	Tan et al. (2015) [63]	The capacity of an individual to mobilise health-sustaining resources from a myriad sources – family, community and culture	To examine the efficacy of a mindfulness-based group intervention for adolescents with mixed mental health disorders	Quantitative Randomized controlled trial	An **outcome** RSCA [94] Global Score
	Marvin et al. (2017) [64]	A set of skills (e.g., social and emotional competencies) that can be taught and/or strengthened	To evaluate the Strong Teens social-emotional learning curriculum among adolescent girls in a residential treatment center	Quantitative Non-equivalent quasi-experimental wait-list control	An **outcome** SEARS [95] Global Score
	Hauber et al. (2019) [65]	The belief that one can cope with stressful life events	To identify therapeutic factors in adolescents’ written narratives, and relate these to changes in symptoms after treatment	Mixed-methods study	A **process** over time, and personal / environmental (therapeutic) **resilience factors**
	Hadebe et al. (2020) [66]	The ability of an individual to function completely in the face of adversity or stress	To explore young adults’ resilience and social support networks	Qualitative Exploratory study	Environmental (social support) **resilience factors**
	Gårdvik et al. (2021) [67]	Positive adaptation to risk exposure, and a more positive psychological outcome than would be expected in case of high levels of environmental adversities	To examine whether resilience factors and treatment procedures among adolescents first presenting at mental health clinics were related to psychiatric symptom load three years later	Quantitative Prospective longitudinal cohort study ^a^SGBA	Personal / environmental **resilience factors** READ [96] Global Scale & 5 Subscales: personal competence, social competence, structured style, family cohesion, social resources
	Zimmermann et al. (2021) [68]	A positive outcome despite adversity	To investigate movement synchrony in relation to therapeutic outcomes during psychotherapy treatment	Quantitative Observational study	Personal / environmental (movement synchrony) **resilience factors**
**Recovery-Focused** **(*n* = 4)** Guiding Frameworks: Recovery models of mental health [97–99], transdiagnostic treatment model [100] Main resilience theorists: Aranda (interpretivist) [101], Luthar (multidimensional) [22, 102], Bottrell (social theory) [103], Richardson (metatheory) [104], Bonanno (recovery vs. resilience distinction) [105, 106] Masten (developmental / multi-systems) [92, 93] Conceptualized resilience as: A dynamic and multidimensional process which promotes functioning and recovery Focus: Adaptive strengths and processes in the context of youth’s recovery Approach: Person-centred	Henderson et al. (2015) [69]	A dynamic process wherein individuals display positive adaptation despite experiences of significant adversity or trauma	To explore young people’s experience of a first episode of psychosis over time and to develop a substantive theory of their responses and behaviors	Qualitative Grounded theory study	A **process** over time
	Las Hayas et al. (2016) [70]	A dynamic process in which psychological, social, environmental, and biological factors interact to enable an individual at any stage of life to develop, maintain, or regain his/her mental health despite exposure to adversity [107]	To explore the role of resilience in recovery from eating disorders (EDs), and to develop a model of resilience in women with EDs	Qualitative Grounded theory study	A **process** over time, and personal / environmental **resilience factors**
	Grob et al. (2020) [71]	A set of complex, subjective processes through which individuals negotiate their complicated journey toward clarified identity and life purpose	To explore how participants' depression impacted their transition from adolescence to emerging adulthood, and built their capacity to form a coherent identity and find a purpose in life	Qualitative Grounded theory study ^b^EDI ^c^Youth engagement (consultation)	A **process** over time
	Luther et al. (2020) [72]	The capacity of a dynamic system to withstand or recover from significant challenges that threaten its stability, viability, or development	To examine whether resilience differs among those with early vs. prolonged psychosis, and the associations between resilience scores and specific symptom domains	Quantitative Cross-sectional study	Personal **resilience factors** RS [84] Global Score & 2 Subscales: personal competence, acceptance of self and life
**Critical and Cultural Perspectives (*n* = 5)** Guiding Frameworks: Social-ecological frameworks (capital theory [108], ecological systems theory [52]) and recovery models of mental health [109, 110] Main resilience theorists: Bottrell (social theory) [103], Ungar (social-ecological) [23], Luthar (multidimensional) [22], Masten (developmental) [92], Richardson (metatheory) [104] Rutter (interactive) [91] Conceptualized resilience as: A process that unfolds over time, dependent on one’s culture and the supportive capacity of the environment Focus: Increased emphasis on service-related factors, systemic barriers, macro-level environment, and culture Approach: Person-centred	Delman et al. (2017) [73]	The employee’s ability to rebound and manage their health in the face of challenges and adversity that affect their work	To examine facilitators of young adult peer provider success in community mental health treatment settings	Qualitative Exploratory study ^c^Youth engagement (partnership)	A **process** over time, personal / environmental **resilience factors**
	Lal et al. (2017) [74]	A process of an individual’s efforts to navigate and negotiate towards resources considered meaningful for well-being in the presence of adversity, and the environment’s concurrent capacity to support individual efforts	To understand how mental health and related services support and hinder resilience in young people diagnosed with first episode psychosis	Qualitative Grounded theory and narrative inquiry ^b^EDI	A **process** over time
	Rayner et al. (2018) [75]	A unique youth recovery process that enabled young people to take on the various challenges in life despite systemic (i.e., lack of employment opportunities or social exclusion) and mental health adversity	To develop a thematic model of youth recovery utilising the experiences of young people with severe mental illness	Qualitative Narrative study	A **process** over time
	Shalanski et al. (2019) [14]	A dynamic process in which positive adaptation is achieved despite significant adversity	To explore resilience from the perspective of teenage girls recovering from mental illness, and to provide an alternative view de- pathologizing psychiatric diagnoses	Qualitative Interpretive phenomenological study	A **process** over time
	Kim et al. (2020) [76]	The dynamic process of adaptation to challenging life conditions that could be protective against mental problems	To investigate the clinical characteristics and psychosocial factors associated with depression in patients with early psychosis according to stage of illness	Quantitative Cross-sectional study ^a^SGBA	Personal **resilience factors** BRS [111] Global Score

*CD-RISC* Connor-Davidson Resilience Scale [83], *RS* The Resilience Scale [84], *RSCA* Resiliency Scales for Children and Adolescents [94], *SEARS* The Social Emotional Assets and Resilience Scale [95], *READ* Resilience Scale for Adolescents [96], *BRS* Brief Resilience Scale [111]

^a^SGBA, sex- and gender-based analyses

^b^EDI, used recruitment strategies to maximize diversity and inclusion among participants

^c^Engaged youth as research advisors or partners

In terms of study designs, fourteen studies (58.3%) were quantitative, nine studies (37.5%) were qualitative, and only one study (4.2%) used a mixed-method design. Two studies used specific recruitment strategies to improve diversity and inclusion among participants, five studies reported sex- and gender-based analyses, and two studies engaged transition-age youth with SMI in the research process. Resilience was operationalized and evaluated as one or more of the following: a set of resilience factors (e.g., risk / vulnerability and protective factors) (58.3%), a mechanism (e.g., mediator or moderator between risks and adaptive outcomes) (8.3%), an outcome (20.8%), or a process that unfolds over time (41.7%).

**Fig. 2:**
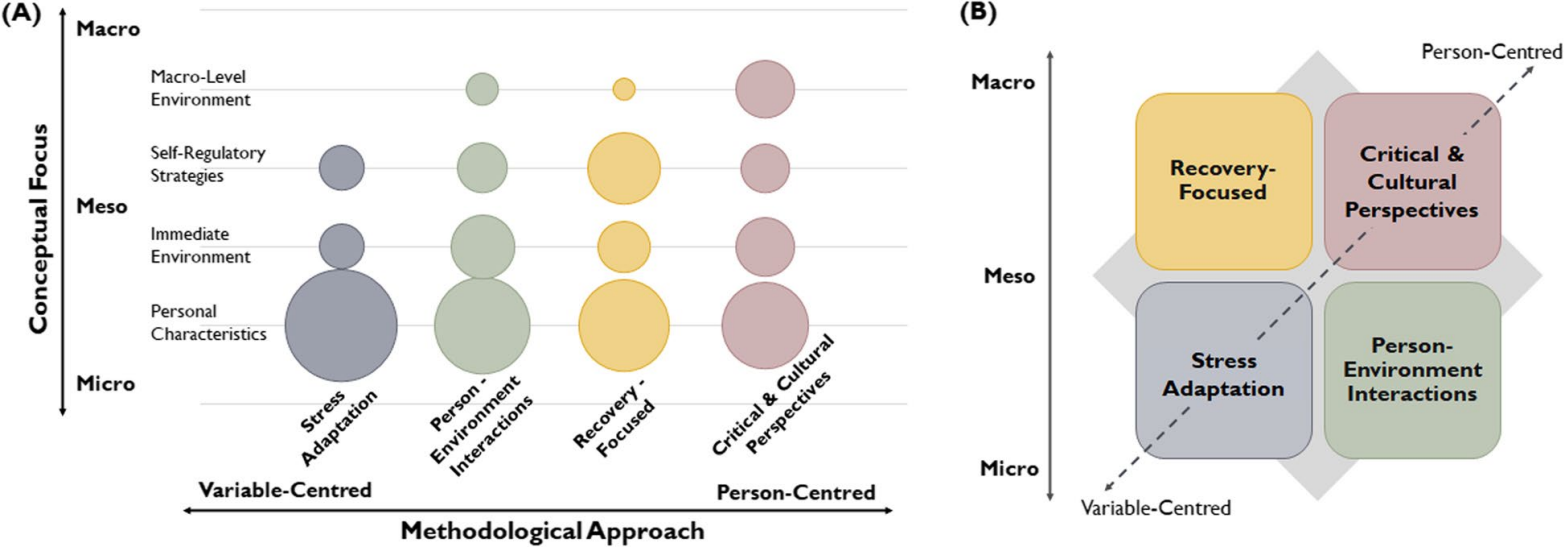
Research traditions. Note: Visual representation of the four research traditions, illustrating main theoretical, conceptual, and methodological trends. **A** Bubble chart. Larger bubbles indicate a greater proportion (%) of resilience factors identified across micro-, meso-, and macrosystems (y-axis) within the respective research tradition (x-axis). **B** Diagram. The solid line (left) represents changes in the theoretical and conceptual focus and the dashed line (diagonal) represents shifts in the predominant methodological approaches employed

Analyses did not elucidate a clear temporal trend based on the publication dates of the articles in each research tradition. However, results indicated that the study of resilience has evolved over time based on: (1) shifts in the main theorists and publication dates of conceptual papers cited (from interactive and developmental perspectives first proposed in the 1990’s, to multidimensional and multi-system perspectives in the early 2000’s, to the most recent social-ecological frameworks that emerged from 2009/2011 onwards); (2) differences in the conceptual focus (from primarily individual-level factors, to the immediate environment and macro-level environment); and (3) changes to the dominant methodological approaches (from variable-centred to person-centred approaches). The research traditions are ordered and described to illustrate these trends, including the theoretical orientations, conceptual focus, and methodological/instrumental approaches. A visual summary is also shown in Fig. 2.

#### Stress adaptation (2012 – 2020)

The Stress Adaptation research tradition included six studies [55–60]. Rutter’s (2012) interactive model [81] and Masten et al.’s (1990) developmental perspective [82] of resilience, as well as integrative models of stress and coping [77–80], were used to emphasize the role of biological, psychological, social, and environmental processes that influence resilience throughout youth development. By integrating resilience theory with stress and coping models, resilience was framed as a process of positive adaptation following stressful or adverse life experiences, and as a protective factor contributing to stress and coping responses. In this tradition, resilience was conceptualized as transition-age youth’s positive adaptation or resistance to stress, which enabled them to maintain their mental health despite exposure to risk. Accordingly, definitions of resilience referred to young people’s ability to adapt, cope and bounce back in response to stress or adversity.

Studies within the Stress Adaptation tradition explored the protective role of resilience factors in the relationship between stressful life experiences and the development or severity of mental illness. Articles also focused on identifying psychological, genetic, and neural markers that may confer resiliency or vulnerability to mental illness. The main outcome of interest was characterized as doing psychologically well (e.g., the absence or reduction of psychopathological symptoms). All six studies within this tradition employed quantitative study designs (e.g., cross-sectional, randomized clinical trial, longitudinal). Resilience was operationalized and evaluated as a set of resilience factors, as a mechanism, and as an outcome. Five studies used validated self-report measures of resilience that included items / subscales to capture personal characteristics reflective of youth’s resilience (e.g., self-efficacy, optimism) [83, 84].

#### Person-environment interactions (2003 – 2021)

Nine studies make up the Person-Environment Interactions research tradition [26, 61–68], which placed more emphasis on how processes of resilience evolve over time and are facilitated through supportive environments. These studies incorporated a wide range of psychosocial theories [85–89] to guide investigations among transition-age youth with SMI. In addition to the interactive [81, 90, 91] and developmental perspectives [82, 92, 93] of resilience found in the Stress Adaptation tradition, the study of resilience was framed through Luthar et al.’s (2000) multidimensional perspective [22], which emphasizes the contribution of both internal and external factors in shaping youth’s response to hardships and individual processes of resilience. Through these guiding frameworks, resilience was conceptualized as a set of transactional processes (between youth and their environment), triggered in response to experiences of adversity. Successful adaptation was still a defining component of resilience, however, environmental factors and person-environment interactions were increasingly recognized.

In this tradition, investigations were centered on understanding reciprocal relationships between the person and the environment, as well as the protective effects of resilience for youth mental health. As such, a broader range of key concepts and outcomes were explored related to the individual (e.g., psychosocial adjustment, symptoms, role functioning) and the immediate environment (e.g., social support networks, relational / therapeutic processes). The use of longitudinal, mixed-method, intervention, and narrative study designs supported the exploration of resilience over the life-course and/or multiple time points. Resilience was operationalized and evaluated as a set of resilience factors, an outcome, and a process. Three studies directly measured resilience using validated scales [94, 95], one of which was designed to evaluate multidimensional components (both personal and environmental) [96].

#### Recovery-focused (2015 – 2020)

The Recovery-Focused research tradition comprised four studies [69–72], which shared many features and theoretical underpinnings with the first two traditions. However, this research tradition explicitly situated the study of resilience within the context of transition-age youth’s mental health recovery. Recovery models [97–99] and transdiagnostic frameworks [100] of mental health were combined with interpretivist [101], multidimensional [22], and social theories [103] of resilience. As such, there was a shift in conceptual papers and theorists that informed the Recovery-Focused tradition, including Luthar et al.’s (2000) and Masten et al.’s (2011) more recent work (e.g., multi-systems perspective) [22, 93, 102], as well as Bonanno [105, 106], Richardson [104], and Bottrell [103]. These frameworks and theorists position resilience as a complex, multidimensional, and subjective process, involving youth’s adaptation and growth following significant adversity. Resilience was conceptualized as both a protective factor and dynamic process that enables youth to develop or regain their mental health, and is thus a critical component of the recovery journey.

Studies within the Recovery-Focused tradition had a unique focus on how resilience unfolds in conjunction with, and in comparison to, processes of recovery. Additionally, researchers aimed to clearly distinguish the concept of resilience from the concept of recovery. From a recovery-oriented lens, studies of resilience included a wider range of key concepts and adaptive outcomes, including experiences of meaning making, re-constructing identity, life purpose, and acceptance. There was also greater recognition of the self-regulatory strategies that young people adopted to bolster their resilience and recovery (e.g., coping skills, help-seeking). Three studies used qualitative grounded theory designs to explore interactions among protective factors and adaptive outcomes, which operationalized resilience as a process. In the one quantitative study, personal resilience factors were evaluated using a validated resilience scale [84].

#### Critical and cultural perspectives (2017 – 2020)

Five studies were included in the final research tradition, Critical and Cultural Perspectives [14, 73–76]. This research tradition emphasized the role of broader societal systems and contexts in shaping resilience and/or critically analyzed the implications of resilience research among transition-age youth with SMI. Similar to the Person-Environment Interactions and Recovery-Focused research traditions, recovery models of mental health [109, 110] were integrated with multidimensional, interactive, and developmental perspectives of resilience [22, 91, 92, 104]. However, Ungar’s (2011) social-ecological resilience framework [23] and Bottrell’s (2009) social resilience theory [103] were at the forefront of this research, which have recently brought greater attention to the cultural context of resilience. Guided by these social-ecological frameworks, resilience was conceptualized as a process that changes over time, facilitates youth’s positive adaptation and recovery, and is dependent on the supportive capacity of one’s environment.

The studies within this research tradition focused on macro-level ecological systems that drive resilience processes, particularly one’s social, cultural, and institutional environment and the quality of resources / services within these contexts. This extended the conceptual focus beyond factors within the individual, family, or immediate environment, and highlighted a wider range of social determinants of health (e.g., stigma, housing, work conditions). Additionally, resilience was studied through a more critical perspective, whereby studies recognized systemic facilitators / barriers to building resilience among transition-age youth living with SMI, and offered a critique and new insights with implications for mental health policies and services (e.g., patient-centred, trauma-informed, gender-responsive, culture-specific services). Four studies within the Critical and Cultural Perspectives tradition used qualitative methods, which operationalized and evaluated resilience as a process. One study employed a quantitative design, evaluating resilience factors using a validated self-report measure [111].

### RQ 2: What factors influence resilience among transition-age youth with SMI, and what outcomes have been studied?

Five core elements of resilience were explored across the 24 included articles. Results are summarized in Table 4. Four types of adversity were characterized as significant sources of challenge or hardship specific to youth with SMI. Analyses revealed that a large breadth of influential resilience factors (personal characteristics, environmental resources, self-regulatory strategies) have been studied. Most research attention has been placed on transition-age youth’s personal characteristics, with a total of 31 unique internal protective / risk factors identified. In contrast, 12 external protective / risk factors were identified as part of youth’s environmental resources, with most belonging within the immediate environment (e.g., family, peers, community) rather than the macro-level environment (e.g., broader societal, cultural, institutional systems) [52]. Ten self-regulatory strategies were identified, explaining the mechanisms and processes that transition-age youth adopted to manage their mental health and build resilience. Twelve resilience-related outcomes were measured or described, spanning indices of mental illness and functioning, to how youth adapted, overcame challenges, and found purpose in life.

**Table 4:** Five core elements of resilience studied among transition-age youth with SMI

Main Category	Subcategories
**Adversity**	
	Onset / experience of SMI [14, 26, 62, 64–67, 69–71, 73–75] Trauma / abuse [14, 26, 55, 59, 61, 62, 76] Difficulty navigating life transitions [62, 69–71] Disconnection from friends, family, or community [26, 62, 64]
**Personal Characteristics (internal protective / risk factors)**	
Psychosocial	Global (trait) resilience [57–59, 65, 67, 70, 72, 73, 75, 76], Functional competence / psychosocial adjustment [26, 62, 65, 67, 72, 75, 76], Perseverance and desire for change [14, 62, 69, 70, 73], Meaning making [62, 71, 74], Attachment style [26, 61, 62] Personality characteristics [26, 61], Turning points [65, 70], Trust [14, 65]
Self & Identity (Cognitive)	Self-beliefs / self-perceptions [14, 55, 62, 71, 73, 75], Self-esteem / self-worth [14, 26, 61, 62, 65, 71], Self-awareness / self-knowledge [14, 62, 65, 70, 71, 75], Sex / gender identity [26, 60, 61, 67, 76], Age [60, 76], Identity formation [71, 75]
Affective	Acceptance [69–72, 75], Hope / optimism [14, 55, 65, 70, 75], Emotional expression [62, 65, 70], Spirituality / autonomy [55, 75]
Behavioral	Responsibility / accountability [14, 65, 70, 75], Substance use [26, 62, 75, 76], Criminality [26, 62], Structured style [67]
Clinical	Mental health disorder / symptoms [26, 56, 58, 62, 67, 71, 76], Stage of illness [72, 76], Duration of untreated mental illness [76]
Physical health	Psychosomatic symptoms [58], Sexual health [76], HRQOL [76], BMI [76]
Biological	Neural factors [56], Genetic factors [60]
**Environmental Resources (external protective / risk factors)**	
Immediate Environment	Social support networks and connectedness, including: Family support / environment [14, 26, 61, 62, 66, 67, 69–71, 73, 75, 76], Peer / interpersonal relationships [14, 26, 61, 62, 65, 66, 69, 73, 75], Professional support [69, 70, 73, 74], Community resources [14, 67] Childhood development and life experiences [14, 56, 61, 75], Informational support [65, 70, 73, 74], Family history of mental illness / suicidal behaviors [14, 56, 61], Relational processes [65, 68]
Macro-Level	Education / employment [26, 61, 73, 75, 76], Stigma / social expectations [14, 71, 73, 75], Family SES / income [61, 75, 76], Accessibility and nature of health services [74, 75], Isolation / alienation [14, 66], Housing [74, 75], Working conditions [73]
**Self-Regulatory Strategies (to self-manage mood, emotions, thoughts, behaviors)**	
	Engagement in services / treatment [57, 60, 63–65, 67–69, 74, 75], Agency & working towards goals [14, 62, 69–71, 75], Coping skills & efforts to increase well-being [14, 69–71], Social / occupational (re)engagement [70, 71, 75], Medication [67, 69, 75], Help-seeking [14, 62, 69], Helping others [65, 71, 73], Communication style [73], Mindfulness [63], Living in the here and now [70]
**Resilience-Related Outcomes**	
	Symptoms of mental illness [55, 57, 59, 60, 63–65, 67, 68, 70, 72] Functioning in valued activities / roles [14, 26, 62, 66, 68, 69, 72, 73] Mental health / well-being [26, 63, 64, 70, 74] Positive adaptation / development [14, 62, 66, 69, 70] Overcoming challenges [14, 62, 66, 69] Global (trait) resilience [57, 60, 63, 64] Presence / absence of mental disorder [55, 56, 59, 76] Personal recovery processes [14, 70, 75] Finding and pursuing purpose in life [69–71] Suicidal ideation / behaviors [58, 61] Therapeutic process [64, 68] Re-constructing identity [71]

*SMI* Serious mental illness, *HRQOL* Health-related quality of life, *BMI* Body mass index, *SES* Socio-economic status

### Consultation with community advisory groups

Four virtual focus groups were conducted to gain the perspectives and feedback of potential knowledge users. Table 5 provides a summary of findings from the consultative meetings with illustrative quotes. Ten transition-age youth aged 20–28 years old who were diagnosed, treated, or living with SMI participated in the topic consultation and reaction meetings (M_age_ = 23.9; 40% White; 70% Women). Most reported a history of multiple co-occurring mental health conditions (50%; e.g., anxiety, mood, personality disorders, PTSD, or schizophrenia spectrum disorders), anxiety (10%), mood disorder (20%), or comorbid anxiety and mood disorders (20%). Ten clinicians involved in the delivery of health services for youth with SMI and researchers in mental health also participated in separate discussions (M_age_ = 32.6 years; 50% White; 80% Women). Clinicians and researchers were from a variety disciplines, primarily occupational therapy (70%), psychology / psychiatry (20%) and rehabilitation sciences (10%), and had diverse experiences working in hospital (50%), community (20%), private practice (20%), and university / academic settings (20%). Most participants joined online from the province of Ontario (*n* = 15), as well as Alberta (*n* = 2), Manitoba (*n* = 1), New Brunswick (*n* = 1), and Quebec (*n* = 1).

**Table 5:** Main categories and subcategories reflecting community advisors’ perspectives and feedback during the review process

Subcategories	Exemplary Quotes (pseudonyms)
**Topic consultation: Perceptions of resilience**	
1. **Resilience is a multidimensional construct, without a single “universal” definition**	
Involves personal strengths and supportive environments	“In terms of resilience in this context, I think of it as like an individual's ability to kind of bounce back from something that um has impacted their mental health in a negative way…the individual's ability to learn from it and then, kind of, become stronger.” – Marlot (youth) “I just feel like resilience is something that is always going to be changing. Like it's going to be affected by the clients' experiences, their cultural backgrounds, their support, their families.” – Eva (clinician)
Difficult to define in real-world contexts	“It's not something that, yeah, like I hear the youth really talk about explicitly… so that might be another part of it… when you're going through the weeds, it can be difficult to kind of name.” – Derrick (clinician)
2. **How resilience is framed matters**	
Buzzword—personal responsibility vs. external systems	“Where um people are constantly in a situation where they *need* to be ‘resilient’… how is our use of resiliency impacting how we frame these people in different situations?” – Jasmine (youth) “It feels a little bit like a backhanded compliment, like ‘oh, but you're so resilient,’ when it is more like, more about larger socioeconomic um systems at play.” – Isabelle (clinician)
**Topic consultation: Main interests and what community advisors most wanted to learn from the review**	
1. **Environmental factors and strategies that influence resilience**	
Role of social determinants of health	“Special consideration should be taken to account about, like race, ethnicity and socioeconomic status, and like other demographics…. that really impacts, I think, resilience… there's multiplied effects of being in multiple minority groups.” – Helen (youth) “Highlighting the importance of cultural groups… *any* of the social determinants of health.” – Isabelle (clinician)
How to improve resilience	“It's kind of about establishing a toolkit. Like what practices are key to resilience? What kind of coping strategies maybe help with resilience and which of them are crucial?” – Zoe (youth) “What about the people that continue to struggle?… what can we learn from this that would help prevent that?” – Derrick (clinician)
2. **How resilience is conceptualized**	
Definitions	“The narrative around resilience… also what you're trying to be resilient from, what you're going through. And I wonder if there is a definition or categories of how that will be defined or framed?” – Jasmine (youth) “I'm curious about how, like, the concept of resiliency has changed in the literature.” – Isabelle (clinician)
Measures and outcomes	“If we learn more about how resilience is defined, or maybe learn about more outcome measures, maybe that will help us, like, clients achieve their goals. Maybe it can help us change our practice.” – Eva (clinician)
**Reaction meeting: Overall impression of the review findings**	
1. **Research traditions and resilience factors that resonated the most with advisors’ personal experiences**	
Person-environment interactions and sociocultural determinants	“I think for me the social determinants of health, they’re such a big factor in everything in your life, but especially you know mental health services… factoring in home situations and homelessness.” – Tina (youth) “It [culture] is going to impact seeking help, accessing resources, knowledge about resources… the impact that it would have with your family so, I think that's a huge consideration.” – Phoebe (clinician)
Youth recovery and self-regulatory strategies	“I thought the recovery focused tradition resonated the most with me… the kind of things that I consume that makes the most difference to my mental health is actually hearing about… how they were able to overcome or get over their mental health issues.” – Yvonne (youth) “Whether the youth accessed mental health resources before and what coping strategies they have like under their belt currently that helps them build resilience… that's kind of what I think about when we talk about the recovery focused tradition.” – Julia (clinician)
Service-related factors	“Sometimes people forget how much of an influence culture has… what works for a certain population of people may not work for somebody else because it's not culturally appropriate… I think that also plays into the service-related factors.” – Katie (youth) “Family is so key… it's often the parents that are calling to ask for resources and supports of how to navigate the system… also not so much access [but] is it readily available in their environment?" – Kirsten (clinician)
2. **How resilience was portrayed in the results**	
Research traditions	“I was wondering if like the… the traditions changed, overtime?” – Yvonne (youth) “How you had the different traditions… kind of, how they build their story… I think that’s great” – Phoebe (clinician)
Process model of resilience	“One of my favorite things about this is just ‘changing symptoms’. It doesn't necessarily mean positive or negative… it's okay to kind of like… just re-go through the process.” – Zoe (youth) “I love this, and I think it's very complex, like I think you have a lot, you've captured a lot here.” – Phoebe (clinician)
**Reaction meeting: Gaps and future research priorities**	
1. **Macro-level environment and patient-oriented research practices**	
Culture and spirituality	“One thing that uh I think a lot of people don't look into, at least from my experience, is religion.” – Katie (youth) “Perspectives from different cultures and countries… that also should be considered here.” – Kirsten (clinician)
Diversity and intersectionality	“Including like an EDI lens would be very important… different religions, races and whatnot… this is something that I am kind of realizing now… as someone who um, kind of faced these barriers.” – Ariel (youth) “Youth resilience research that looks at different marginalized groups…. for example, um, youth living with disabilities… youth who are from the LGBTQ + community… different underserved groups. That's kind of front and center in my mind.” – Kirsten (clinician)
Youth and community engagement	“Going into the community and actually asking what they want, rather than assuming… see what they need… have the community involved in that process. Making sure that it's aligning um with their values.” – Ariel (youth)
2. **Transdiagnostic resilience factors over the life-course**	
Co-occurring diagnoses or mental health issues	“We should think more about how different mental health issues occur together and interact with one-another, since it's not uncommon for people to have more than one issue at the same time.” – Yvonne (youth)
Physical health	“I do think sometimes um, like, psychosomatic symptoms are overlooked, um and even just the way your physical health can degrade as a result of your mental illness or… side effects of medications.” – Zoe (youth)
Developmental stages and life transitions	“During the different life transitions… people have different resources and support, and resilience looks different if you're 13 versus if you're, you know, 24.” – Lena (clinician)
**Knowledge dissemination preferences and applicability**	
1. **Combining visual and written summaries**	
Figures / models	“I think a combination of a diagram for visual learners, and also written summary.” – Eva (clinician) “Infographics are more accessible for lay audiences.” – Helen (youth)
2. **Application**	
Knowledge translation tool	“I feel like this is so useful and meaningful to so many people, and especially if it's in a really digestible form… like ‘Oh check out this thing… it shows a really comprehensive um kind of overview’… that would be really great… it makes it really accessible.” – Zoe (youth) “Presenting families and um clients with something like similar to this model… I'm really interested in like the wave at the bottom… do you think showing them… and explaining how it comes from research might help them in their resiliency journey?” – Cara (researcher)
Tangible resources or programming	“Resiliency training has been popping up and could help in different format options.” – Tina (youth) “Programs that could be offered, partnerships that could be pursued with different community organizations… Like who's operating in the ecosystem and who can we connect with to bridge gaps… to just be stronger together in different communities.” – Kirsten (clinician)

#### Perceptions of resilience

Resilience was described by participants as multidimensional, involving both personal strengths and supportive environments that enable people to bounce back after adversities. Particularly, resilience was viewed as both a trait (e.g., internal strength or characteristic) and a process, in that it changes throughout the life-course in response to personal circumstances. Relatedly, participants highlighted that resilience is difficult to define in real-world contexts, as experiences and meanings of resilience for youth are highly personal. In this way, participants underscored the plurality and subjectivity of resilience, expressing that there are multiple ways to define and understand this complex concept (as opposed to a single, universal definition). Concerns were also raised regarding the “weaponization” of resilience. Participants elaborated on the use of the term “resilience” as a buzzword, which from their perspective emphasizes the individual’s role or responsibility in overcoming trauma and adversity, over and beyond the health and social systems that shape resilience processes through the provision of external resources and opportunities. As such, how resilience is framed in research and practice was noted as an important consideration.

#### Main areas of interest in the study of resilience

Identifying environmental factors and strategies that contribute to youth resilience was expressed as a key interest. Participants suggested that understanding the role of social determinants of health in shaping resilience processes would be valuable information, including the population characteristics and demographics of youth who have been involved in resilience research (e.g., race, culture, socioeconomic status). Specific coping skills and health care practices that have been shown to enhance resilience were also highlighted as important areas of focus that may inform future targets for intervention. Additionally, participants expressed interest in understanding how resilience has been conceptualized, including definitions, measurement, and resilience-related outcomes. Taken together, perceptions of resilience and main interests gathered in the topic consultation meetings aligned with the focus and scope of the review. Participant feedback was applied to refine the charting form and analyses.

#### Overall impression and results that resonated the most

In the reaction meetings, person-environment interactions were discussed in relation to how clinicians identify protective factors in practice and the influence of youth’s social support network. Relatedly, participants highlighted the importance of cultural and social determinants of health in how young people subjectively experience resilience and navigate the mental health care system. The recovery-focused tradition and self-regulatory strategies resonated as well. Participants emphasized resilience-related outcomes centered on adaptation and overcoming challenges as particularly meaningful, as this better acknowledged the ebb and flow of clinical symptoms, recovery-oriented practices and resources, and non-linear pathways of resilience. Self-regulatory strategies stood out among participants, as these results demonstrated the practical and tangible ways that young people build resilience and cope with the challenges of SMI. Interestingly, the most salient review findings were continuously linked to participants’ perspectives of youth mental health services. This underscored the critical role of service-related factors that contribute to processes of resilience. Clinicians and researchers highlighted the role of family-centred services and timely access to support, and youth participants emphasized the importance of culturally appropriate services. Lastly, the use of research traditions to share evolving storylines and trends, as well as a resilience model (figure) visually summarizing the core elements of resilience, were expressed as favorable ways to communicate the results. Collectively, discussions revealed that the review findings resonated with participants’ personal experiences and practices.

#### Gaps and future research priorities

Focus group discussions led to two key areas recommended for future research. First, increased focus on the macro-level environment and patient-oriented research was suggested. Specifically, participants identified a need for more research that considers transition-age youth’s culture and spirituality, as well as the importance of diversity and intersectionality (e.g., understanding resilience among marginalized and diverse populations with intersecting social identities). Youth participants also recommended engaging youth and community members within research activities as a priority to advance resilience research among transition-age youth with SMI. Second, the identification of developmental and transdiagnostic resilience factors among youth with SMI was proposed as a future research direction (e.g., resilience factors, mechanisms and processes that are shared across multiple diagnoses and developmental periods). Youth participants emphasized the importance of understanding resilience factors that are relevant across diagnoses of SMI given the high prevalence of co-occurring mental health conditions. This included greater recognition of biological and physical health indicators, which transition-age youth described as an often overlooked area that is relevant to their resilience and mental health recovery (e.g., psychosomatic symptoms, medication side effects). In contrast, clinicians and researchers highlighted the importance of further exploring similarities and differences in how resilience unfolds according to different life stages and transitions.

#### Knowledge dissemination preferences and applicability

Participants had a preference for both written and visual summaries to synthesize information. Participants expressed that a figure or model that captures resilience processes among transition-age youth with SMI would support knowledge translation among peers, colleagues, and clients. Additionally, participants suggested that the results of the review may be applied to inform the development of tangible resources and programming aimed at promoting resilience among transition-age youth experiencing SMI. In sum, consultative meetings suggested that the results of the present review have practical value for informing future research and practices in youth mental health care. Participant feedback informed the final reporting of the scoping review results.

## Discussion

The present scoping review provides a comprehensive synthesis of resilience research among transition-age youth with SMI, while integrating community advisory group feedback. Four research traditions emerged, each portraying processes of resilience through a unique storyline: Stress Adaptation, Person-Environment Interactions, Recovery-Focused, Critical and Cultural Perspectives. Resilience factors and outcomes were most commonly evaluated at the individual level or within the immediate environment, with fewer studies exploring the interplay of cultural processes, contexts, and broader societal systems. Community advisors shared the extent to which these results reflected their personal views, knowledge, and practices. Based on the perspectives of transition-age youth, clinicians and researchers, the findings of this review may inform directions for future research and advance practices within resilience-informed care approaches.

### Current tensions within and across the research traditions

Tensions arose across the included articles in how resilience was conceptualized and investigated as a process. For inclusion in this scoping review, articles had to adopt a process-oriented perspective, which acknowledges that resilience is a changing state influenced by both internal strengths and the environmental resources that are afforded to youth [20, 21]. Yet, many studies did not adopt methodological approaches that allowed for evaluations of resilience over time, and only one study used a validated resilience measure that included subscales capturing environmental resilience factors [67]. Similar tensions and discrepancies have been highlighted in prior reviews of resilience research among youth and adult populations [17, 18, 30–34, 112]. As noted by these authors, variability in how resilience is framed and defined has contributed to significant challenges in the measurement of resilience, the interpretation of study comparisons, and current understandings of resilience-informed interventions in research and clinical practice. There is currently no gold standard for self-report measures of resilience for those undertaking quantitative studies [113], and the extent to which existing measures apply to youth living with SMI is not well understood [114].

Also similar to prior reviews of resilience research [17, 18, 30–34, 112], inconsistencies were observed in the terminology used across studies (e.g., resilience vs. resiliency; protective factors vs. resilience factors; interactions vs. transactions), which can lead to confusion and ambiguity [31]. Researchers are encouraged to provide a clear and explicit definition of resilience, which logically flows to the study purpose and rationale for methodological choices, instruments or techniques [31, 32]. The key theorists and conceptual papers detailed across the four research traditions identified in this review may provide a starting point for the selection and use of consistent language in future work.

Importantly, the four research traditions described in the present review overlapped and showed similarities in definitions and measures of resilience. This is expected in meta-narrative reviews synthesizing a complex field of study, where scientific evidence continuously integrates and builds on past knowledge [36]. However, by mapping how resilience has been studied across different research traditions, additional tensions were brought to light. Conceptualizations and operationalizations of resilience among transition-age youth with SMI varied, particularly through shifts in the guiding theorists and frameworks, which coincided with changes in the conceptual focus and methodologies – from predominantly variable-centred to more person-centred approaches. Variable-centred approaches often use quantitative designs to investigate the link between risk and protective factors to adaptive outcomes, as well as targetable mechanisms that may buffer the impact of risks / adversity on one’s health and development [18, 112, 115]. In contrast, person-centred approaches often use longitudinal or qualitative methods to delineate how resilience unfolds for specific individuals, enabling evaluations and comparisons of resilience over time, individual lived experiences, and contexts [18, 112, 115]. With the uptake of more advanced statistical analyses (e.g., multilevel modelling, network analysis) and mixed-method study designs, researchers can leverage the strengths of both variable-centred and person-centred approaches in studies of resilience among transition-age youth with SMI [18, 116, 117].

Prior reviews spanning multiple areas of study and populations have highlighted trends in how resilience research has evolved over time, which parallel the tensions seen here [18, 30]. Particularly, these changes and shifts were similarly reflected as four “waves” of resilience research [118–120], which have progressed from a focus on personal protective factors, to explorations of socio-cultural influences and interventions, to the most recent multi-system perspective that aims to promote multiple levels of analysis and cross-disciplinary research. Khanlou and Wray (2014) [30] have also synthesized these shifts in their review of resilience literature and outline three approaches: a focus on individual factors, a constructionist approach, an ecological and ecosystemic approach.

Consultative meetings with community advisory groups confirmed the potential value of organizing resilience research in a way that portrays unique research traditions or ‘storylines’. Particularly, the research traditions described herein demonstrate evolutions in thinking which may be used to guide future theory-driven research with strong epistemological congruence. Thus, results of the current review may contribute to enhancing conceptual clarity within the study of resilience among young people with SMI.

### Resilience factors and outcomes

**Fig. 3:**
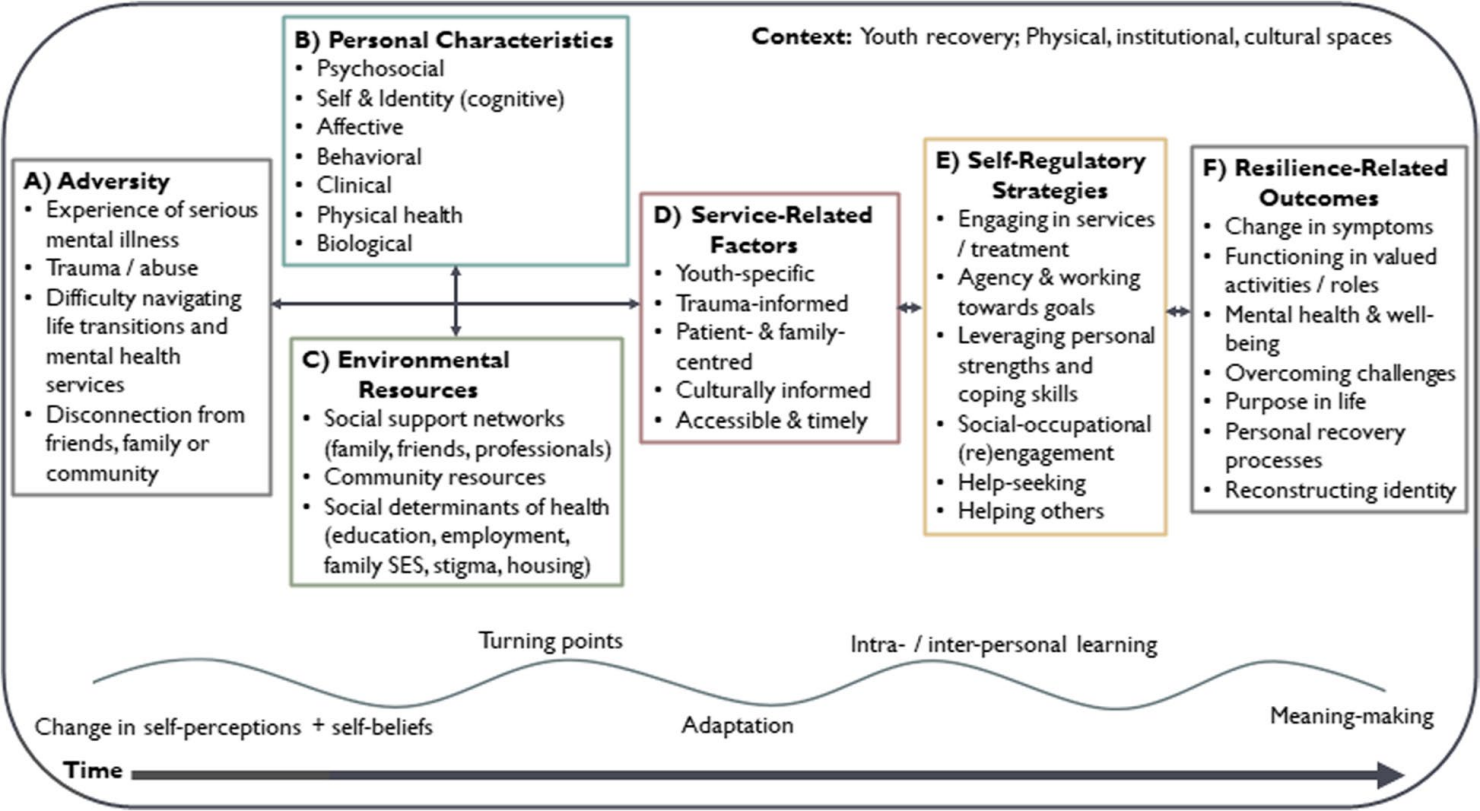
Process model of resilience among transition-age youth with SMI. Note: Processes of resilience are depicted through person-in-environment transactions. Core elements include: (**A**) experiences of adversity; multi-modal resilience factors: (**B**) personal characteristics, (**C**) environmental resources, (**D**) service-related factors, (**E**) self-regulatory strategies; and (**F**) resilience-related outcomes. Resilience processes are illustrated as unfolding over time, through non-linear pathways based on one’s subjective experiences, and bound within youth’s context. B & C: Results of the present scoping review suggest that personal (internal) and environmental (external) factors may exist along a continuum of risk and protective effects. This model was adapted from current process-oriented models of resilience in rehabilitation sciences [20, 21]

The current review also uncovered how five core elements of resilience have been characterized within the transition-age youth mental health literature. Findings revealed that a broad scope of resilience factors and outcomes have been studied. Overall, the results resonated with community advisors’ personal experiences and perspectives of resilience. However, the central role of supportive and accessible health services in fostering resilience among transition-age youth with SMI was uniquely highlighted in the review findings, and equally emphasized in the consultative meetings. Transition-age youth who have experienced SMI, clinicians, and researchers called attention to the importance of contextualizing resilience processes and focusing on service-related factors and practices that may support youth’s resilience. These results were used to expand on the process-oriented models of resilience which informed the present study [20, 21], with the addition of “service-related factors” as a central component. A process model of resilience developed in collaboration with transition-age youth, clinicians, and researchers is shown in Fig. 3 to clearly depict the main findings and key messages.

To date, the greatest amount of research attention has been placed on identifying resilience factors assessed at the individual level. The personal characteristics identified largely overlapped with internal risk and protective factors that have been highlighted in the adult mental health and developmental psychopathology literature [17, 18, 30–32, 34]. Also consistent with prior resilience research, biological and physical health characteristics were rarely explored as influential resilience factors among transition-age youth with SMI. This is somewhat surprising considering the well-known physical health risks associated with SMI, and emerging therapeutic practices targeting healthy lifestyle behaviors and mind–body connection [121, 122]. The self-regulatory strategies identified also mirror prior research [18, 32, 114, 123], and highlighted the importance of engaging in supportive services, coping skills, youth’s agency in working towards goals and participating in meaningful occupations. Results demonstrate a variety of potential mechanisms of change that can be further explored and expanded on in efforts to support processes of resilience in young people with SMI.

Importantly, the resilience factors listed above are inseparable from individuals’ environmental resources and opportunities [25]. Within the context of young people’s mental health recovery, researchers have increasingly emphasized the importance of understanding how resilience processes arise through person-in-environment transactions, which are contextually and culturally dependent [9, 30, 31, 123, 124]. As evidenced by the results of the current review, and consistent with prior research [17, 18, 30–32, 34], most studies have considered environmental resources and transactional processes that involve youth’s immediate environment (e.g., social support networks). The role of broader social, political, institutional, and cultural systems that makeup the macro-level environment can be much more difficult to investigate, and has therefore received less attention [30, 35]. Relatedly, findings demonstrated that there is currently limited resilience research among transition-age youth with SMI belonging to racialized, marginalized, or other underserved groups. Increased focus on the macro-level environment and experiences of youth with diverse and intersecting social identities would reveal important social and contextual factors that impact health inequalities, and in turn influence meanings and processes of resilience [25, 30, 35].

Experiences of adversity and positive adaptation are considered defining features of resilience that are closely linked [8, 9]. The present review revealed that transition-age youth with SMI face unique challenges and adversities, which underscores the importance of trauma-informed care approaches among this population [14]. Future research is warranted that fully contextualizes various types of adversity during this transitional period of development [35]. Further, enhancing access and engagement in trauma-specific evidence-based treatment should be considered in future research and clinical practice, as this may be a central resource for supporting positive cascading effects in the resilience processes of transition-age youth living with SMI [14, 18, 114]. Similar to prior reviews of resilience research [31, 34], clinical outcomes designed to assess pathology and impairment were still a common focus. However, findings illustrated a clear trend towards exploring positive outcomes that reflect youth’s adaptation, functioning, well-being and life purpose, which more closely align with strengths-based and recovery-oriented approaches [8, 9, 17, 123, 124]. There is ongoing debate in resilience research as to what exactly should be considered a positive resilience-related outcome [8, 112]. The perspectives of transition-age youth who have experienced SMI, members of their social support network, and mental health professionals should be prioritized to identify outcomes that matter the most and build on the results of this review [32, 35, 114].

Taken together, the present review synthesized a wide scope of resilience factors and outcomes that have been studied among transition-age youth with SMI. Results extended prior process-oriented models of resilience by identifying factors related to the provision of health services as a central component unique to this population. Further, by uncovering resilience factors and outcomes shown to be salient across and beyond specific diagnoses of SMI, results can be used to inform the design and delivery of services catered to a broader range of youth mental health service users. As such, results hold practical implications and provide valuable insights into protective factors, mechanisms, and transdiagnostic intervention targets that may support youth’s positive development, functioning, and well-being.

### Future research directions

Based on the scoping review findings, including community advisory group consultations, five key areas are recommended for future resilience research among transition-age youth with SMI:

1. Interdisciplinary and integrative studies are needed to build a knowledge base that is relevant to researchers and clinicians from various disciplines.
2. Intersectional approaches and collaborative research practices focused on fostering youth and community engagement are needed to gain the perspectives of more diverse populations and bring co-produced knowledge to the forefront of future work.
3. Mixed-method and longitudinal study designs, as well as advanced statistical analyses, should be applied in future research. These methods have the capacity to examine dynamic processes of resilience and interactions between multi-modal resilience factors.
4. Further investigations of developmental and transdiagnostic resilience factors are needed to clarify how resilience unfolds during different life stages and resilience factors that are shared within and across multiple SMI diagnoses (including physical / biological markers).
5. Macro-level environmental factors which impact resilience processes among transition-age youth with SMI should be a focus of future resilience research to understand broader social and cultural determinants that may inform health policy changes. In addition to the research designs and methods stated above, future work may consider adopting specific strategies suggested for exploring the influence of broader contexts, systems, and health services on youth resilience processes, such as: participatory / co-design research approaches, prioritizing contextually and culturally relevant outcomes, greater consideration of social determinants of health within the main data analyses and reporting [35, 114, 125, 126], and the use of resilience measures capable of evaluating social-ecological resources (e.g., community inclusion and opportunities, cultural identity, spirituality) [96, 127, 128].

### Strengths and limitations

There are several limitations to the present review which should be noted. First, an assessment of the methodological quality of evidence was not completed as this is beyond the scope of a scoping review design [42, 43, 47]. The lack of quality assessment limits the types of conclusions and implications that can be drawn from the current results [43]. Second, variability in how the population (transition-age youth) and concept (resilience) have been defined, as well as restrictions to the search strategy based on language, date, and publication type may have limited the breadth of the search and contributed to English language bias. Relatedly, articles that did not clearly define or operationalize resilience were excluded. As such, there are sources not included in the present review that may still be quite informative to the study of resilience among transition-age youth with SMI (e.g., review papers, interventions that did not define resilience).

The application of recent guidelines for high quality and transparent reporting is a notable strength to the current review which helped to mitigate the challenges and limitations mentioned above [44, 45, 48, 54]. Additionally, the scoping review protocol was developed and carried out by a multidisciplinary review team with backgrounds in occupational therapy / rehabilitation sciences (AEN, SPB, EJN), kinesiology (MLdJ, CMS), and psychiatry / early intervention (NK). The integration of multiple perspectives and academic disciplines supported the study selection process and interpretation of the review findings. Lastly, the inclusion of community advisory group consultations was a key strength of the present review. This is a crucial step that has been recommended for advancing resilience research [35] and as part of the scoping review methodology [43, 46] to promote a more collaborative approach and emphasize the voices of young people and knowledge users. Incorporating the perspectives and feedback of transition-age youth who have experienced SMI, as well as mental health clinicians and researchers, was essential for maximizing the relevance and overall contribution of the research.

## Conclusions

The distinct impact and burden of SMI among young people has been increasingly recognized among researchers and clinicians. Results of the present scoping review demonstrate that investigations of resilience among transition-age youth with SMI are growing in popularity, and hold strong potential for revealing novel strengths and resources that can inform the development of innovative youth mental health practices and policies. Further research is encouraged that adopts interdisciplinary and intersectional approaches, and prioritizes community and youth engagement in research practices, in order to deepen current understandings of resilience among transition-age youth with SMI.

### Supplementary Information

**Additional file 1:** **Supplementary File 1.** PRISMA-ScR Checklist.

**Additional file 2:** **Supplementary File 2.** Complete multi-database online search strategy conducted Dec 6, 2021.

## Data Availability

The dataset generated / analyzed as part of the review are included in this published article (and its supplementary information files). The qualitative focus group data that supports the review findings are potentially identifiable and thus not suitable for sharing via a public database. The data are available from the corresponding author upon reasonable request (subject to ethical permissions and participant consent).

## Abbreviations

BMI: Body mass index
BRS: Brief Resilience Scale
CD-RISC: Connor-Davidson Resilience Scale
Dx: Diagnosis
EDI: Strategies to maximize diversity and inclusion among participants
HRQOL: Health-related quality of life
PCC: Population, concept, context
PRISMA-ScR: Preferred Reporting Items for Systematic reviews and Meta-Analyses extension for Scoping Reviews Checklist
READ: Resilience Scale for Adolescents
RS: The Resilience Scale
RSCA: Resiliency Scales for Children and Adolescents
SEARS: The Social Emotional Assets and Resilience Scale
SES: Socio-economic status
SGBA: Sex- and gender-based analyses
SMI: Serious mental illness

## References

[1] McGorry P (2011). Transition to adulthood: The critical period for pre-emptive, disease-modifying care for schizophrenia and related disorders. Schizophr Bull.

[2] Pearson C, Janz T, Ali J. Mental and substance use disorders in Canada. Health at a Glance. Statistics Canada. Catalogue no. 82–624-X. 2013.

[3] Arnett JJ, Žukauskienė R, Sugimura K (2014). The new life stage of emerging adulthood at ages 18–29 years: Implications for mental health. Lancet Psychiatry.

[4] Stoep AV, Beresford SA, Weiss NS, McKnight B, Cauce AM, Cohen P (2000). Community-based study of the transition to adulthood for adolescents with psychiatric disorder. Am J Epidemiol.

[5] Fusar-Poli P. Integrated mental health services for the developmental period (0 to 25 years): A critical review of the evidence. Front Psychiatry. 2019;10. 10.3389/fpsyt.2019.00355. 10.3389/fpsyt.2019.00355 PMC6567858 31231250

[6] Yung AR, Cotter J, McGorry PD. Youth mental health: Approaches to emerging mental ill-Health in young people. London: Routledge; 2020.

[7] Fletcher D, Sarkar M (2013). Psychological resilience: A review and critique of definitions, concepts, and theory. Eur Psychol.

[8] Stainton A, Chisholm K, Kaiser N, Rosen M, Upthegrove R, Ruhrmann S (2019). Resilience as a multimodal dynamic process. Early Interv Psychiatry.

[9] Echezarraga A, Las Hayas C, López de Arroyabe E, Jones SH. Resilience and recovery in the context of psychological disorders. J Humanist Psychol. 2019; 10.1177/0022167819851623

[10] Mental Health Commission of Canada (2021). Changing directions, changing lives: The mental health strategy for Canada.

[11] Ministry of Health and Long-Term Care (2011). Respect, recovery, resilience: Recommendations for Ontario’s mental health & addictions strategy.

[12] World Health Organization. Mental health action plan 2013–2020. 2013. Available from: https://apps.who.int/iris/handle/10665/89966

[13] Friesen BJ (2007). Recovery and resilience in children’s mental health: Views from the field. Psychiatr Rehabil J.

[14] Shalanski L, Ewashen C (2019). An interpretive phenomenological study of recovering from mental illness: Teenage girls’ portrayals of resilience. Int J Ment Health Nurs.

[15] Fusar-Poli P, Solmi M, Brondino N, Davies C, Chae C, Politi P (2019). Transdiagnostic psychiatry: A systematic review. World Psychiatry.

[16] Fritz J, de Graaff AM, Caisley H, van Harmelen A-L, Wilkinson PO. A systematic review of amenable resilience factors that moderate and/or mediate the relationship between childhood adversity and mental health in young people. Front Psychiatry. 2018;9. 10.3389/fpsyt.2018.00230. 10.3389/fpsyt.2018.00230 PMC6018532 29971021

[17] Davydov DM, Stewart R, Ritchie K, Chaudieu I (2010). Resilience and mental health. Clin Psychol Rev.

[18] Masten AS, Lucke CM, Nelson KM, Stallworthy IC (2021). Resilience in development and psychopathology: Multisystem perspectives. Annu Rev Clin Psychol.

[19] Fine SB (1991). Resilience and human adaptability: Who rises above adversity?. Am J Occup Ther.

[20] McLarnon MJW, Rothstein MG (2013). Development and initial validation of the workplace resilience inventory. J Pers Psychol.

[21] Nalder E, Hartman L, Hunt A, King G (2019). Traumatic brain injury resiliency model: A conceptual model to guide rehabilitation research and practice. Disabil Rehabil.

[22] Luthar SS, Cicchetti D, Becker B (2000). The construct of resilience: A critical evaluation and guidelines for future work. Child Dev.

[23] Ungar M (2011). The social ecology of resilience: Addressing contextual and cultural ambiguity of a nascent construct. Am J Orthopsychiatry.

[24] Hutcheon E, Lashewicz B (2014). Theorizing resilience: Critiquing and unbounding a marginalizing concept. Disabil Soc.

[25] Ungar M, Theron L (2020). Resilience and mental health: How multisystemic processes contribute to positive outcomes. Lancet Psychiatry.

[26] Gralinski-bakker JH, Hauser ST, Stott C, Billings RL, Allen JP, Hauser ST (2004). Markers of resilience and risk: Adult lives in a vulnerable population. Res Hum Dev.

[27] Browne J, Estroff SE, Ludwig K, Merritt C, Meyer-Kalos P, Mueser KT (2018). Character strengths of individuals with first episode psychosis in Individual Resiliency Training. Schizophr Res.

[28] Browne J, Mueser KT, Meyer-Kalos P, Gottlieb JD, Estroff SE, Penn DL (2019). The therapeutic alliance in individual resiliency training for first episode psychosis: Relationship with treatment outcomes and therapy participation. J Consult Clin Psychol.

[29] Schwarz S (2018). Resilience in psychology: A critical analysis of the concept. Theory Psychol.

[30] Khanlou N, Wray R (2014). A whole community approach toward child and youth resilience promotion: A review of resilience literature. Int J Ment Health Addict.

[31] Rudzinski K, McDonough P, Gartner R, Strike C. Is there room for resilience? A scoping review and critique of substance use literature and its utilization of the concept of resilience. Subst Abus Treat Prev Policy. 2017;12(1). 10.1186/s13011-017-0125-2 10.1186/s13011-017-0125-2 PMC5603070 28915841

[32] Christmas CM, Khanlou N (2019). Defining youth resilience: A scoping review. Int J Ment Health Addict.

[33] Raanaas RK, Bjøntegaard H, Shaw L (2020). A scoping review of participatory action research to promote mental health and resilience in youth and adolescents. Adolesc Res Rev.

[34] Tankersley AP, Grafsky EL, Dike J, Jones RT (2021). Risk and resilience factors for mental health among transgender and gender nonconforming (TGNC) youth: A systematic review. Clin Child Fam Psychol Rev.

[35] Hart A, Gagnon E, Eryigit-Madzwamuse S, Cameron J, Aranda K, Rathbone A, et al. Uniting resilience research and practice with an inequalities approach. SAGE Open. 2016;6(4). 10.1177/2158244016682477

[36] Greenhalgh T, Robert G, Macfarlane F, Bate P, Kyriakidou O, Peacock R (2005). Storylines of research in diffusion of innovation: A meta-narrative approach to systematic review. Soc Sci Med.

[37] United Nations. Definition of youth. 2013. Available from: http://www.un.org/ esa/socdev/documents/youth/fact-sheets/youth-defnition.pdf.

[38] McGorry P, Bates T, Birchwood M (2013). Designing youth mental health services for the 21st century: Examples from Australia, Ireland and the UK. Br J Psychiatry.

[39] National Institute of Mental Health. Mental illness 2021. Available from: https://www.nimh.nih.gov/health/statistics/mental-illness.shtml#:~:text=Serious mental illness (SMI) is,or more major life activities

[40] Substance Abuse and Mental Health Services Administration. Behind the term: Serious mental illness. Development Services Group, Inc. 2016. Available from: https://www.hsdl.org/?abstract&did=801613

[41] Nesbitt AE, Sabiston CM, deJonge ML, Barbic SP, Kozloff N, Nalder EJ (2022). Understanding resilience among transition-age youth with serious mental illness: Protocol for a scoping review. BMJ Open..

[42] Arksey H, O’Malley L (2005). Scoping studies: Towards a methodological framework. Int J Soc Res Methodol.

[43] Levac D, Colquhoun H, O’Brien KK (2010). Scoping studies: Advancing the methodology. Implement Sci.

[44] Tricco AC, Lillie E, Zarin W, O’Brien KK, Colquhoun H, Levac D (2018). PRISMA Extension for Scoping Reviews (PRISMA-ScR): Checklist and Explanation. Ann Intern Med.

[45] Wong G, Greenhalgh T, Westhorp G, Buckingham J, Pawson R (2013). RAMESES publication standards: Meta-narrative reviews. J Adv Nurs.

[46] Sabiston CM, Vani M, DeJonge M, Nesbitt A. Scoping reviews and rapid reviews. Int Rev Sport Exerc Psychol. 2022;15(1). 10.1080/1750984X.2021.1964095

[47] Munn Z, Peters MDJ, Stern C, Tufanaru C, McArthur A, Aromataris E (2018). Systematic review or scoping review? Guidance for authors when choosing between a systematic or scoping review approach. BMC Med Res Methodol.

[48] McGowan J, Sampson M, Salzwedel DM, Cogo E, Foerster V, Lefebvre C (2016). PRESS peer review of electronic search strategies: 2015 guideline statement. J Clin Epidemiol.

[49] Darnay K, Hawke LD, Chaim G, Henderson JL, INNOVATE Research Team (2019). INNOVATE Research: Youth Engagement Guidebook for Researchers.

[50] Elo S, Kyngäs H (2008). The qualitative content analysis process. J Adv Nurs.

[51] Elo S, Kääriäinen M, Kanste O, Pölkki T, Utriainen K, Kyngäs H. Qualitative content analysis: A focus on trustworthiness. SAGE Open. 2014;4(1). 10.1177/2158244014522633

[52] Bronfenbrenner U (1979). The Ecology of Human Development: Experiments by Nature and Design.

[53] Smith B, McGannon KR (2018). Developing rigor in qualitative research: Problems and opportunities within sport and exercise psychology. Int Rev Sport Exerc Psychol.

[54] Anderson JK, Howarth E, Vainre M, Humphrey A, Jones PB, Ford TJ (2020). Advancing methodology for scoping reviews: Recommendations arising from a scoping literature review (SLR) to inform transformation of Children and Adolescent Mental Health Services. BMC Med Res Methodol.

[55] Seok JH, Lee KU, Kim W, Lee SH, Kang EH, Ham BJ (2012). Impact of early-life stress and resilience on patients with major depressive disorder. Yonsei Med J.

[56] Fischer AS, Camacho MC, Ho TC, Whitfield-Gabrieli S, Gotlib IH (2018). Neural markers of resilience in adolescent females at familial risk for major depressive disorder. JAMA Psychiat.

[57] Konradt CE, de Cardoso T A, Mondin TC, de Souza LD M, Kapczinski F, da Silva RA (2018). Impact of resilience on the improvement of depressive symptoms after cognitive therapies for depression in a sample of young adults. Trends Psychiatry Psychother..

[58] De Berardis D, Fornaro M, Valchera A, Rapini G, Di Natale S, De Lauretis I (2020). Alexithymia, resilience, somatic sensations and their relationships with suicide ideation in drug naïve patients with first-episode major depression: An exploratory study in the “real world” everyday clinical practice. Early Interv Psychiatry.

[59] Vieira IS, Pedrotti Moreira F, Mondin TC, de Cardoso T A, Branco JC, Kapczinski F (2020). Resilience as a mediator factor in the relationship between childhood trauma and mood disorder: A community sample of young adults. J Affect Disord..

[60] Peters RB, Xavier J, Mondin TC, de Cardoso T A, Ferreira FB, Teixeira L (2021). Bdnf val66met polymorphism and resilience in major depressive disorder: The impact of cognitive psychotherapy. Brazilian J Psychiatry..

[61] Fergusson DM, Beautrais AL, Horwood LJ (2003). Vulnerability and resiliency to suicidal behaviours in young people. Psychol Med.

[62] Hauser ST, Allen JP (2007). Overcoming adversity in adolescence: Narratives of resilience. Psychoanal Inq.

[63] Tan L, Martin G (2015). Taming the adolescent mind: A randomised controlled trial examining clinical efficacy of an adolescent mindfulness-based group programme. Child Adolesc Ment Health.

[64] Marvin LA, Caldarella P, Young EL, Young KR (2017). Implementing Strong Teens for adolescent girls in residential treatment: A quasi-experimental evaluation. Resid Treat Child Youth.

[65] Hauber K, Boon AE, Vermeiren R (2019). Therapeutic factors that promote recovery in high-risk adolescents intensive group psychotherapeutic MBT programme. Child Adolesc Psychiatry Ment Health.

[66] Hadebe NF, Ramukumba TS (2020). Resilience and social support of young adults living with mental illness in the city of Tshwane, Gauteng province. South Africa Curationis.

[67] Gårdvik KS, Rygg M, Torgersen T, Wallander JL, Lydersen S, Indredavik MS (2021). Association of treatment procedures and resilience to symptom load three-years later in a clinical sample of adolescent psychiatric patients. BMC Psychiatry.

[68] Zimmermann R, Fürer L, Kleinbub JR, Ramseyer FT, Hütten R, Steppan M (2021). Movement synchrony in the psychotherapy of adolescents with borderline personality pathology – A dyadic trait marker for resilience?. Front Psychol.

[69] Henderson AR, Cock A (2015). The responses of young people to their experiences of first-episode psychosis: Harnessing resilience. Community Ment Health J.

[70] Las Hayas C, Padierna JA, Muñoz P, Aguirre M, del GómezBarrio A, Beato-Fernández L (2016). Resilience in eating disorders: A qualitative study. Women Heal..

[71] Grob R, Schlesinger M, Wise M, Pandhi N (2020). Stumbling into adulthood: Learning from depression while growing up. Qual Health Res.

[72] Luther L, Rosen C, Cummins JS, Sharma RP (2020). The multidimensional construct of resilience across the psychosis spectrum: Evidence of alterations in people with early and prolonged psychosis. Psychiatr Rehabil J.

[73] Delman J, Klodnick VV (2017). Factors Supporting the employment of young adult peer providers: Perspectives of peers and supervisors. Community Ment Health J.

[74] Lal S, Ungar M, Malla A, Leggo C, Suto M (2017). Impact of mental health services on resilience in youth with first episode psychosis: A qualitative study. Adm Policy Ment Heal Ment Heal Serv Res.

[75] Rayner S, Thielking M, Lough R (2018). A new paradigm of youth recovery: Implications for youth mental health service provision. Aust J Psychol.

[76] Kim SW, Kim JJ, Lee BJ, Yu JC, Lee KY, Won SH (2020). Clinical and psychosocial factors associated with depression in patients with psychosis according to stage of illness. Early Interv Psychiatry.

[77] McEwen BS (2013). The brain on stress: Toward an integrative approach to brain, body and behavior. Perspect Psychol Sci.

[78] Wu G, Feder A, Cohen H, Kim JJ, Calderon S, Charney DS, et al. Understanding resilience. Front Behav Neurosci. 2013;7. 10.3389/fnbeh.2013.00010. 10.3389/fnbeh.2013.00010 PMC3573269 23422934

[79] Charney DS (2004). Psychobiological mechanisms of resilience and vulnerability: Implications for successful adaptation to extreme stress. Am J Psychiatry.

[80] Feder A, Nestler EJ, Charney DS (2009). Psychobiology and molecular genetics of resilience. Nat Rev Neurosci.

[81] Rutter M (2012). Resilience as a dynamic concept. Dev Psychopathol.

[82] Masten AS, Best KM, Garmezy N (1990). Resilience and development: Contributions from the study of children who overcome adversity. Dev Psychopathol.

[83] Connor KM, Davidson JRT (2003). Development of a new resilience scale: The Connor-Davidson Resilience Scale (CD-RISC). Depress Anxiety.

[84] Wagnild GM, Young HM (1993). Development and psychometric evaluation of the Resilience Scale. J Nurs Meas.

[85] Charon R (2001). Narrative medicine: A model for empathy, reflection, profession, and trust. JAMA.

[86] Payton JW, Wardlaw DM, Graczyk PA, Bloodworth MR, Tompsett CJ, Weissberg RP (2000). Social and emotional learning: A framework for promoting mental health and reducing risk behavior in children and youth. J Sch Health.

[87] Joiner TE, Steer RA, Beck AT, Schmidt NB, Rudd MD, Catanzaro SJ (1999). Physiological hyperarousal: Construct validity of a central aspect of the tripartite model of depression and anxiety. J Abnorm Psychol.

[88] Yalom I, Leszcz M (2005). The theory and practice of group psychotherapy.

[89] Feldman R (2020). What is resilience: an affiliative neuroscience approach. World Psychiatry.

[90] Rutter M (1987). Psychosocial resilience and protective mechanisms. Am J Orthopsychiatry.

[91] Rutter M (2006). Implications of resilience concepts for scientific understanding. Ann N Y Acad Sci.

[92] Masten AS (2001). Ordinary magic: Resilience processes in development. Am Psychol.

[93] Masten AS (2011). Resilience in children threatened by extreme adversity: Frameworks for research, practice, and translational synergy. Dev Psychopathol.

[94] Prince-Embury S (2006). Resiliency scales for children and adolescent: Profiles of personal strengths.

[95] Merrell KW (2011). SEARS: Social emotional assets and resilience scales.

[96] Hjemdal O, Friborg O, Stiles TC, Martinussen M, Rosenvinge JH (2006). A new scale for adolescent resilience: Grasping the central protective resources behind healthy development. Meas Eval Couns Dev.

[97] Henderson AR (2011). A substantive theory of recovery from the effects of severe persistent mental illness. Int J Soc Psychiatry.

[98] Frese FJ, Stanley J, Kress K, Vogel-Scibilia S (2001). Integrating evidence-based practices and the recovery model. Psychiatr Serv.

[99] Chiu MYL, Ho WWN, Lo WTL, Yiu MGC (2010). Operationalization of the SAMHSA model of recovery: A quality of life perspective. Qual Life Res.

[100] Fairburn CG, Cooper Z, Shafran R (2003). Cognitive behaviour therapy for eating disorders: A “transdiagnostic” theory and treatment. Behav Res Ther.

[101] Aranda K, Zeeman L, Scholes J, Morales AS-M (2012). The resilient subject: Exploring subjectivity, identity and the body in narratives of resilience. Heal An Interdiscip J Soc Study Heal Illn Med..

[102] Luthar SS, Cicchetti D (2000). The construct of resilience: Implications for interventions and social policies. Dev Psychopathol.

[103] Bottrell D (2009). Understanding ‘marginal’ perspectives: Towards a social theory of resilience. Qual Soc Work.

[104] Richardson GE (2002). The metatheory of resilience and resiliency. J Clin Psychol.

[105] Bonanno GA (2004). Loss, trauma, and human resilience: Have we underestimated the human capacity to thrive after extremely aversive events?. Am Psychol.

[106] Bonanno GA (2005). Resilience in the face of potential trauma. Curr Dir Psychol Sci.

[107] Wathen CN, MacGregor JC, Hammerton J, Coben JH, Herrman H, Stewart DE (2012). Priorities for research in child maltreatment, intimate partner violence and resilience to violence exposures: Results of an international Delphi consensus development process. BMC Public Health.

[108] Bliss C, Cohen AJ, Harcourt GC (2005). Capital Theory:.

[109] Anthony WA (1993). Recovery from mental illness: The guiding vision of the mental health service system in the 1990s. Psychosoc Rehabil J.

[110] Leamy M, Bird V, Le BC, Williams J, Slade M (2011). Conceptual framework for personal recovery in mental health: Systematic review and narrative synthesis. Br J Psychiatry.

[111] Smith BW, Dalen J, Wiggins K, Tooley E, Christopher P, Bernard J (2008). The brief resilience scale: Assessing the ability to bounce back. Int J Behav Med.

[112] Windle G (2011). What is resilience? A review and concept analysis. Rev Clin Gerontol.

[113] Windle G, Bennett KM, Noyes J (2011). A methodological review of resilience measurement scales. Health Qual Life Outcomes.

[114] Thompson EG, Knowles SF, Greasley P (2019). Understanding resilience in young people with complex mental health needs: A Delphi study. Clin Child Psychol Psychiatry.

[115] Luthar SS, Cushing G, Glantz M, Johnson JL (1999). Measurement issues in the empirical study of resilience: An overview. Resilience and Development: Positive Life Adaptations.

[116] Kalisch R, Cramer AOJ, Binder H, Fritz J, Leertouwer IJ, Lunansky G (2019). Deconstructing and reconstructing resilience: A dynamic network approach. Perspect Psychol Sci..

[117] Fritz J, Fried EI, Goodyer IM, Wilkinson PO, van Harmelen AL (2018). A network model of resilience factors for adolescents with and without exposure to childhood adversity. Sci Rep.

[118] Masten AS (2007). Resilience in developing systems: Progress and promise as the fourth wave arises. Dev Psychopathol.

[119] Ungar M, Brown M, Liebenberg L, Othman R, Kwong WM, Armstrong M (2008). Unique pathways to resilience across cultures. Youth Stud Aust.

[120] Wright M, Masten A, Narayan A, Goldstein S, Brooks R (2013). Handbook of Resilience in Children. Handbook of Resilience in Children.

[121] Firth J, Solmi M, Wootton RE, Vancampfort D, Schuch FB, Hoare E (2020). A meta-review of “lifestyle psychiatry”: The role of exercise, smoking, diet and sleep in the prevention and treatment of mental disorders. World Psychiatry.

[122] Vancampfort D, Stubbs B, Van Damme T, Smith L, Hallgren M, Schuch F (2021). The efficacy of meditation-based mind-body interventions for mental disorders: A meta-review of 17 meta-analyses of randomized controlled trials. J Psychiatr Res.

[123] Zautra A, Hall J, Murray K, the Resilience Solutions Group (2008). Resilience: A new integrative approach to health and mental health research. Health Psychol Rev..

[124] Liebenberg L (2020). Reconsidering interactive resilience processes in mental health: Implications for child and youth services. J Community Psychol.

[125] Ungar M (2015). Practitioner review: Diagnosing childhood resilience – a systemic approach to the diagnosis of adaptation in adverse social and physical ecologies. J Child Psychol Psychiatry.

[126] Ungar M, Theron L, Murphy K, Jefferies P (2021). Researching multisystemic resilience: A sample methodology. Front Psychol.

[127] Liebenberg L, Ungar M, Vijver FV (2012). Validation of the child and youth resilience measure-28 (CYRM-28) among Canadian youth. Res Soc Work Pract.

[128] Liebenberg L, Moore JC (2018). A social ecological measure of resilience for adults: The RRC-ARM. Soc Indic Res.

