# Supplementary material for: A scoping review of resilience among transition-age youth with serious mental illness: tensions, knowledge gaps, and future directions
Source: BMC Psychiatry. 2023 Sep 7;23:660. doi: 10.1186/s12888-023-05158-0 (PMC10483804; doi:10.1186/s12888-023-05158-0)
Supplement: Supplementary file 2 — Additional file 2: Supplementary File 2. Complete multi-database online search strategy conducted Dec 6, 2021. [file 12888_2023_5158_MOESM2_ESM.docx]

**Supplementary File 2: Complete multi-database online search strategy conducted Dec 6, 2021**

**Medline Database Search Strategy**

| **Search line #** | **PCC conceptual term of interest** | **Search term entered into OVID-MEDLINE** |
| --- | --- | --- |
| 1 | Population (Transition-age youth) | exp Adolescent/ or exp Young Adult/ |
| 2 | Population (Transition-age youth) | (youth* or transition age youth* or teen* or adolescen* or emerging adult* or young adult* or early adult* or young person* or young people* or juvenile*).tw,kf. |
| 3 | Population (Serious mental illness) | exp Mental Disorders/ or exp Anxiety Disorders/ or exp “Bipolar and Related Disorders”/ or exp Dissociative Disorders/ or exp “Feeding and Eating Disorders”/ or exp Mood Disorders/ or exp “Attention Deficit and Disruptive Behavior Disorders”/ or exp Personality Disorders/ or exp Schizophrenia/ or exp Psychotic Disorders/ or exp Affective Disorders, Psychotic/ or exp Capgras Syndrome/ or exp Delusional Parasitosis/ or exp Morgellons Disease/ or exp Paranoid Disorders/ or exp Somatoform Disorders/ or exp “Trauma and Stressor Related Disorders”/ or exp Mentally Ill Persons/ |
| 4 | Population (Serious mental illness) | (mental disorder* or mental illness* or psychiatric disabilit* or psychiatric disorder* or psychiatric diagnosis* or serious emotional disturbance* or severe emotional disturbance* or “mental health condition*” or anxiety disorder* or phobia* or phobic disorder* or panic disorder* or obsessive-compulsive disorder* or OCD or bipolar disorder* or manic disorder* or manic depression or dissociative disorder* or multiple-personality disorder or eating disorder* or anorexi* or bulimi* or binge eating* or “eating disorder not otherwise specified” or EDNOS or “other specified feeding or eating disorder” or OSFED or disordered eating or mood disorder* or depressive disorder* or affective disorder* or depression or cyclothymic disorder* or cyclothymia or dysthymic disorder* or dysthymia or personality disorder* or schizophrenia spectrum disorder* or schizophrenia or psychotic disorder* or psychosis or psychoses or schizoaffective disorder* or psychotic affective disorder* or paranoid disorder* or somatoform disorder* or body dysmorphic disorder* or body dysmorphi* or post-traumatic stress disorder* or adjustment disorder* or PTSD).tw,kf. |
| 5 | Concept (Resilience) | exp Resilience, Psychological/ |
| 6 | Concept (Resilience) | (resilienc*).tw,kf. |
| 7 |  | 1 or 2 |
| 8 |  | 3 or 4 |
| 9 |  | 5 or 6 |
| 10 |  | 7 and 8 and 9 |
| 11 |  | limit 10 to (english language and humans and yr="2000 - Current") |

**Embase Database Search Strategy**

| **Search line #** | **PCC conceptual term of interest** | **Search term entered into OVID-EMBASE** |
| --- | --- | --- |
| 1 | Population (Transition-age youth) | exp Adolescent/ or exp Young Adult/ |
| 2 | Population (Transition-age youth) | (youth* or transition age youth* or teen* or adolescen* or emerging adult* or young adult* or early adult* or young person* or young people* or juvenile*).tw,kf. |
| 3 | Population (Serious mental illness) | exp Mental Disease/ or exp Anxiety Disorder/ or exp Bipolar Disorder/ or exp Dissociative Disorder/ or exp Eating Disorder/ or exp Emotional Disorder/ or exp Mood Disorder/ or exp Attention Deficit Disorder/ or exp Impulse Control Disorder/ or exp Neurosis/ or exp Personality Disorder/ or exp Schizophrenia Spectrum Disorder/ or exp Schizophrenia/ or exp Psychosis/ or exp Somatoform Disorder/ or exp Posttraumatic Stress Disorder/ |
| 4 | Population (Serious mental illness) | (mental disorder* or mental illness* or psychiatric disabilit* or psychiatric disorder* or psychiatric diagnosis* or serious emotional disturbance* or severe emotional disturbance* or “mental health condition*” or anxiety disorder* or phobia* or phobic disorder* or panic disorder* or obsessive-compulsive disorder* or OCD or bipolar disorder* or manic disorder* or manic depression or dissociative disorder* or multiple-personality disorder or eating disorder* or anorexi* or bulimi* or binge eating* or “eating disorder not otherwise specified” or EDNOS or “other specified feeding or eating disorder” or OSFED or disordered eating or mood disorder* or depressive disorder* or affective disorder* or depression or cyclothymic disorder* or cyclothymia or dysthymic disorder* or dysthymia or personality disorder* or schizophrenia spectrum disorder* or schizophrenia or psychotic disorder* or psychosis or psychoses or schizoaffective disorder* or psychotic affective disorder* or paranoid disorder* or somatoform disorder* or body dysmorphic disorder* or body dysmorphi* or post-traumatic stress disorder* or adjustment disorder* or PTSD).tw,kf. |
| 5 | Concept (Resilience) | exp Psychological Resilience/ |
| 6 | Concept (Resilience) | (resilienc*).tw,kf. |
| 7 |  | 1 or 2 |
| 8 |  | 3 or 4 |
| 9 |  | 5 or 6 |
| 10 |  | 7 and 8 and 9 |
| 11 |  | limit 10 to (english language and humans and yr="2000 - Current") |

**PsychINFO Database Search Strategy**

| **Search line #** | **PCC conceptual term of interest** | **Search term entered into OVID-PsycINFO** |
| --- | --- | --- |
| 1 | Population (Transition-age youth) | exp Emerging Adulthood/ or exp Early Adolescence/ |
| 2 | Population (Transition-age youth) | (youth* or transition age youth* or teen* or adolescen* or emerging adult* or young adult* or early adult* or young person* or young people* or juvenile*).tw. |
| 3 | Population (Serious mental illness) | exp Mental Disorders/ or exp Chronic Mental Illness or exp Serious Mental Illness/ or exp Affective Disorders or exp Anxiety Disorders/ or exp Attention Deficit Disorder/ or exp Bipolar Disorder/ or exp Dissociative Disorders/ or exp Disruptive Behavior Disorders/ or exp Eating Disorders/ or exp Personality Disorders/ or exp Psychosis/ or exp Schizophrenia/ or exp Somatoform Disorders/ or exp “Stress and Trauma Related Disorders”/ |
| 4 | Population (Serious mental illness) | (mental disorder* or mental illness* or psychiatric disabilit* or psychiatric disorder* or psychiatric diagnosis* or serious emotional disturbance* or severe emotional disturbance* or “mental health condition*” or anxiety disorder* or phobia* or phobic disorder* or panic disorder* or obsessive-compulsive disorder* or OCD or bipolar disorder* or manic disorder* or manic depression or dissociative disorder* or multiple-personality disorder or eating disorder* or anorexi* or bulimi* or binge eating* or “eating disorder not otherwise specified” or EDNOS or “other specified feeding or eating disorder” or OSFED or disordered eating or mood disorder* or depressive disorder* or affective disorder* or depression or cyclothymic disorder* or cyclothymia or dysthymic disorder* or dysthymia or personality disorder* or schizophrenia spectrum disorder* or schizophrenia or psychotic disorder* or psychosis or psychoses or schizoaffective disorder* or psychotic affective disorder* or paranoid disorder* or somatoform disorder* or body dysmorphic disorder* or body dysmorphi* or post-traumatic stress disorder* or adjustment disorder* or PTSD).tw. |
| 5 | Concept (Resilience) | exp “Resilience (Psychological)”/ |
| 6 | Concept (Resilience) | (resilienc*).tw. |
| 7 |  | 1 or 2 |
| 8 |  | 3 or 4 |
| 9 |  | 5 or 6 |
| 10 |  | 7 and 8 and 9 |
| 11 |  | limit 10 to (english language and humans and yr="2000 - Current") |

**AMED Database Search Strategy**

| **Search line #** | **PCC conceptual term of interest** | **Search term entered into OVID-AMED** |
| --- | --- | --- |
| 1 | Population (Transition-age youth) | exp Adolescent/ |
| 2 | Population (Transition-age youth) | (youth* or transition age youth* or teen* or adolescen* or emerging adult* or young adult* or early adult* or young person* or young people* or juvenile*).tw,et. |
| 3 | Population (Serious mental illness) | exp Mental Disorders/ or exp Adjustment Disorders/ or exp Affective Disorders/ or exp Affective Disorders Psychotic/ or exp Bipolar Disorder/ or exp Manic Disorder/ or exp Depressive Disorder/ or exp Mood Disorders/ or exp Anxiety Disorders/ or exp Obsessive Compulsive Disorder/ or exp Phobic Disorders/ or exp Stress Disorders Post Traumatic/ or exp Attention Deficit Disorder with Hyperactivity or exp Child Behavior Disorders/ or exp Dissociative Disorders/ or exp Multiple Personality Disorder/ or exp Eating Disorders/ or exp Anorexia Nervosa/ or exp Bulimia/ or exp Neurotic Disorders/ or exp Personality Disorders/ or exp Borderline Personality Disorder/ or exp Hysteria/ or exp Psychotic Disorders/ or exp Schizophrenia/ or exp Somatoform Disorders/ or exp Conversion Disorder/ |
| 4 | Population (Serious mental illness) | (mental disorder* or mental illness* or psychiatric disabilit* or psychiatric disorder* or psychiatric diagnosis* or serious emotional disturbance* or severe emotional disturbance* or “mental health condition*” or anxiety disorder* or phobia* or phobic disorder* or panic disorder* or obsessive-compulsive disorder* or OCD or bipolar disorder* or manic disorder* or manic depression or dissociative disorder* or multiple-personality disorder or eating disorder* or anorexi* or bulimi* or binge eating* or “eating disorder not otherwise specified” or EDNOS or “other specified feeding or eating disorder” or OSFED or disordered eating or mood disorder* or depressive disorder* or affective disorder* or depression or cyclothymic disorder* or cyclothymia or dysthymic disorder* or dysthymia or personality disorder* or schizophrenia spectrum disorder* or schizophrenia or psychotic disorder* or psychosis or psychoses or schizoaffective disorder* or psychotic affective disorder* or paranoid disorder* or somatoform disorder* or body dysmorphic disorder* or body dysmorphi* or post-traumatic stress disorder* or adjustment disorder* or PTSD).tw,et. |
| 5 | Concept (Resilience) | exp Adaptation Psychological/ |
| 6 | Concept (Resilience) | (resilienc*).tw,et. |
| 7 |  | 1 or 2 |
| 8 |  | 3 or 4 |
| 9 |  | 5 or 6 |
| 10 |  | 7 and 8 and 9 |
| 11 |  | limit 10 to (english and yr="2000 -Current") |

**CINHAL Database Search Strategy**

| **Search line #** | **PCC conceptual term of interest** | **Search term entered into CINHAL (EBSCO)** |
| --- | --- | --- |
| 1 | Population (Transition-age youth) | (MH “Adolescence+”) OR (MH “Young Adult”) |
| 2 | Population (Transition-age youth) | TI (youth* or “transition age youth*” or teen* or adolescen* or “emerging adult*” or “young adult*” or “early adult*” or “young person*” or “young people*” or juvenile*) OR AB (youth* or “transition age youth*” or teen* or adolescen* or “emerging adult*” or “young adult*” or “early adult*” or “young person*” or “young people*” or juvenile*) |
| 3 | Population (Serious mental illness) | (MH “Mental Disorders+”) or (MH “Mental Disorders, Chronic”) or (MH “Neurotic Disorders+”) or (MH “Affective Disorders+”) or (MH “Seasonal Affective Disorder”) or (MH “Depression+”) or (MH “Anxiety Disorders+”) or (MH “Social Anxiety Disorders”) or (MH “Generalized Anxiety Disorder”) or (MH “Panic Disorder”) or (MH “Obsessive-Compulsive Disorder+”) or (MH “Phobic Disorders+”) or (MH “ Stress Disorders, Post-Traumatic+”) or (MH “Psychotic Disorders+”) or (MH “Schizophrenia+”) or (MH “Affective Disorders, Psychotic+”) or (MH “Bipolar Disorders+”) or  (MH “Dissociative Disorders+”) or (MH “Multiple-Personality Disorder”) or (MH “Personality Disorders+”) or (MH “Adjustment Disorders+”) or (MH “Attention Deficit Hyperactivity Disorder”) or (MH “Child Behavior Disorders+”) or (MH “Eating Disorders+”) or (MH “Binge Eating Disorder”) or (MH “Avoidant Restrictive Food Intake Disorder”) or (MH “Bulimia Nervosa”) or (MH “Bulimia”) or (MH “Anorexia Nervosa”) or (MH “Anorexia”) or (MH “Somatoform Disorders+”) or (MH “Body Dysmorphic Disorder”) |
| 4 | Population (Serious mental illness) | TI (“mental disorder*” or “mental illness*” or “psychiatric disabilit*” or “psychiatric disorder*” or “psychiatric diagnosis*” or “serious emotional disturbance*” or “severe emotional disturbance*” or “mental health condition*” or “anxiety disorder*” or phobia* or “phobic disorder*” or “panic disorder*” or “obsessive-compulsive disorder*” or OCD or “bipolar disorder*” or “manic disorder*” or “manic depression” or “dissociative disorder*” or “multiple-personality disorder” or “eating disorder*” or anorexi* or bulimi* or “binge eating*” or “eating disorder not otherwise specified” or EDNOS or “other specified feeding or eating disorder” or OSFED or “disordered eating” or “mood disorder*” or “depressive disorder*” or “affective disorder*” or depression or “cyclothymic disorder*” or cyclothymia or “dysthymic disorder*” or dysthymia or “personality disorder*” or “schizophrenia spectrum disorder*” or schizophrenia or “psychotic disorder*” or psychosis or psychoses or “schizoaffective disorder*” or “psychotic affective disorder*” or “paranoid disorder*” or “somatoform disorder*” or “body dysmorphic disorder*” or “body dysmorphi*” or “post-traumatic stress disorder*” or “adjustment disorder*” or PTSD) OR AB (“mental disorder*” or “mental illness*” or “psychiatric disabilit*” or “psychiatric disorder*” or “psychiatric diagnosis*” or “serious emotional disturbance*” or “severe emotional disturbance*” or “mental health condition*” or “anxiety disorder*” or phobia* or “phobic disorder*” or “panic disorder*” or “obsessive-compulsive disorder*” or OCD or “bipolar disorder*” or “manic disorder*” or “manic depression” or “dissociative disorder*” or “multiple-personality disorder” or “eating disorder*” or anorexi* or bulimi* or “binge eating*” or “eating disorder not otherwise specified” or EDNOS or “other specified feeding or eating disorder” or OSFED or “disordered eating” or “mood disorder*” or “depressive disorder*” or “affective disorder*” or depression or “cyclothymic disorder*” or cyclothymia or “dysthymic disorder*” or dysthymia or “personality disorder*” or “schizophrenia spectrum disorder*” or schizophrenia or “psychotic disorder*” or psychosis or psychoses or “schizoaffective disorder*” or “psychotic affective disorder*” or “paranoid disorder*” or “somatoform disorder*” or “body dysmorphic disorder*” or “body dysmorphi*” or “post-traumatic stress disorder*” or “adjustment disorder*” or PTSD) |
| 5 | Concept (Resilience) | (MH “Hardiness”) |
| 6 | Concept (Resilience) | TI (resilienc*) OR AB (resilienc*) |
| 7 |  | 1 or 2 |
| 8 |  | 3 or 4 |
| 9 |  | 5 or 6 |
| 10 |  | 7 and 8 and 9 |
| 11 |  | limit 10 to (english language and humans and yr="2000 - Current") |

**Scopus Database Search Strategy**

| **Search line #** | **PCC conceptual term of interest** | **Search term entered into Scopus** |
| --- | --- | --- |
| 1 | Population (Transition-age youth) | TITLE-ABS-KEY (youth* or “transition age youth*” or teen* or adolescen* or “emerging adult*” or “young adult*” or “early adult*” or “young person*” or “young people*” or juvenile*) |
| 2 | Population (Serious mental illness) | TITLE-ABS-KEY (“mental disorder*” or “mental illness*” or “psychiatric disabilit*” or “psychiatric disorder*” or “psychiatric diagnosis*” or “serious emotional disturbance*” or “severe emotional disturbance*” or “mental health condition*” or “anxiety disorder*” or phobia* or “phobic disorder*” or “panic disorder*” or “obsessive-compulsive disorder*” or OCD or “bipolar disorder*” or “manic disorder*” or “manic depression” or “dissociative disorder*” or “multiple-personality disorder” or “eating disorder*” or anorexi* or bulimi* or “binge eating*” or “eating disorder not otherwise specified” or EDNOS or “other specified feeding or eating disorder” or OSFED or “disordered eating” or “mood disorder*” or “depressive disorder*” or “affective disorder*” or depression or “cyclothymic disorder*” or cyclothymia or “dysthymic disorder*” or dysthymia or “personality disorder*” or “schizophrenia spectrum disorder*” or schizophrenia or “psychotic disorder*” or psychosis or psychoses or “schizoaffective disorder*” or “psychotic affective disorder*” or “paranoid disorder*” or “somatoform disorder*” or “body dysmorphic disorder*” or “body dysmorphi*” or “post-traumatic stress disorder*” or “adjustment disorder*” or PTSD) |
| 3 | Concept (Resilience) | TITLE-ABS-KEY (resilienc*) |
| 4 |  | 1 and 2 and 3 |
| 5 |  | limit 4 to (english language and yr="2000 - Current") |
